# GLIS1 regulates trabecular meshwork function and intraocular pressure and is associated with glaucoma in humans

**DOI:** 10.1038/s41467-021-25181-7

**Published:** 2021-08-12

**Authors:** K. Saidas Nair, Chitrangda Srivastava, Robert V. Brown, Swanand Koli, Hélène Choquet, Hong Soon Kang, Yien-Ming Kuo, Sara A. Grimm, Caleb Sutherland, Alexandra Badea, G. Allan Johnson, Yin Zhao, Jie Yin, Kyoko Okamoto, Graham Clark, Terete Borrás, Gulab Zode, Krishnakumar Kizhatil, Subhabrata Chakrabarti, Simon W. M. John, Eric Jorgenson, Anton M. Jetten

**Affiliations:** 1 Department of Ophthalmology and Department of Anatomy, School of Medicine, University of California, San Francisco, San Francisco, CA USA; 2 Immunity, Inflammation and Disease Laboratory, National Institute of Environmental Health Sciences, National Institutes of Health, Research Triangle Park, NC USA; 3 Department of Ophthalmology, School of Medicine, University of California, San Francisco, San Francisco, CA USA; 4 Kaiser Permanente Northern California, Division of Research, Oakland, CA USA; 5 Integrative Bioinformatics Support Group, National Institute of Environmental Health Sciences, National Institutes of Health, Research Triangle Park, NC USA; 6 Center for In Vivo Microscopy, Department of Radiology, Duke University, Durham, NC USA; 7 The Jackson Laboratory, Bar Harbor, ME USA; 8 Department of Ophthalmology, University of North Carolina School of Medicine, Chapel Hill, NC USA; 9 Department of Pharmacology and Neuroscience, North Texas Eye Research Institute, University of North Texas Health Science Center, Fort Worth, TX USA; 10 Brien Holden Eye Research Centre, L. V. Prasad Eye Institute, Hyderabad, India; 11 Howard Hughes Medical Institute, Mortimer B. Zuckerman Mind Brain Behavior Institute, Department of Ophthalmology, Columbia University, New York, NY USA; 12 Regeneron Pharmaceuticals, Inc, Tarrytown, NY USA

**Keywords:** Gene expression, Genetic association study, Transcriptional regulatory elements, Glaucoma

## Abstract

Chronically elevated intraocular pressure (IOP) is the major risk factor of primary open-angle glaucoma, a leading cause of blindness. Dysfunction of the trabecular meshwork (TM), which controls the outflow of aqueous humor (AqH) from the anterior chamber, is the major cause of elevated IOP. Here, we demonstrate that mice deficient in the Krüppel-like zinc finger transcriptional factor GLI-similar-1 (GLIS1) develop chronically elevated IOP. Magnetic resonance imaging and histopathological analysis reveal that deficiency in GLIS1 expression induces progressive degeneration of the TM, leading to inefficient AqH drainage from the anterior chamber and elevated IOP. Transcriptome and cistrome analyses identified several glaucoma- and extracellular matrix-associated genes as direct transcriptional targets of GLIS1. We also identified a significant association between *GLIS1* variant rs941125 and glaucoma in humans (*P* = 4.73 × 10^−6^), further supporting a role for *GLIS1* into glaucoma etiology. Our study identifies GLIS1 as a critical regulator of TM function and maintenance, AqH dynamics, and IOP.

## Abstract (web summary)

Dysfunction of the trabecular meshwork (TM) is the chief cause of elevated intraocular pressure, the major risk factor of glaucoma. Here, the authors identify the transcription factor, GLIS1, as a critical regulator of TM maintenance and intraocular pressure, and as a glaucoma risk gene.

## Introduction

A glaucoma is a heterogeneous group of progressive optic neuropathies characterized by the degeneration of the optic nerve that results in irreversible blindness^1^. Primary open-angle glaucoma (POAG) and primary angle-closure glaucoma (PACG) are the two most common forms of glaucoma in adults^2–5^, while primary congenital glaucoma accounts for up to 18% of childhood blindness^6^. Age, ethnicity, gender, environmental, and genetic factors all contribute to glaucoma susceptibility^7–9^. However, elevated intraocular pressure (IOP) is the major causal risk factor for glaucoma.

Normal IOP is required to maintain the proper physiological function of the eye and also to maintain the structure of the globe of the eye. The maintenance of homeostatic IOP is critically dependent on the balance between the inflow and outflow of aqueous humor (AqH)^10^. AqH is secreted by the ciliary body into the ocular anterior chamber (AC) where it nourishes avascular tissues. The AqH subsequently exits through specialized drainage structures located at the junction where the iris meets the cornea (iridocorneal angle). The ocular drainage structures are primarily composed of the trabecular meshwork (TM) and Schlemm’s canal (SC). AqH first flows through the TM into the SC and subsequently enters the episcleral veins before returning back to the systemic circulation^10–12^. TM dysfunction has been causally linked to impaired AqH drainage (increased outflow resistance) and elevated IOP^10,13–15^.

An increasing number of rare mutations and common genetic variants in a variety of genes, including *MYOC*, *CYP1B1*, *GLIS3*, *LOXL4*, *LTBP2*, *PITX2*, and *OPTN*, have been associated with elevated IOP and different types of glaucoma^4,5,9,16–24^.

GLI-Similar 1 (GLIS1), together with GLIS2 and −3, comprise a subfamily of Krüppel-like zinc finger (ZF) transcriptional factors^25–27^. In contrast to GLIS2 and GLIS3, relatively little is known about the physiological functions of GLIS1. To obtain greater insights into the biological roles of GLIS1, we analyzed *Glis1*-KO mice for phenotypic alterations and found that these mice develop an enlarged eye phenotype.

In this study, we examine the function of GLIS1 in ocular tissues in more detail and demonstrate that GLIS1 plays a critical regulatory role in maintaining normal TM structure and IOP. We show that GLIS1 is expressed in the TM, a tissue critical in the regulation of outflow resistance. Deficiency in *GLIS1* induces progressive degeneration of the TM, leading to inefficient AqH drainage and elevated IOP. To obtain insights into potential mechanisms that may underlie this phenotype, changes in the expression of target genes were examined. Combined RNA-Seq and ChIP-Seq analyses identified a number of genes that are directly regulated by GLIS1, including *MYOC, CYP1B1, LOXL4*, and *LTBP2*, genes previously implicated in glaucoma^6,28^. Importantly, we have detected significant associations between common genetic variants in the *GLIS1* region and POAG in humans, thereby supporting the role of *GLIS1* as a glaucoma risk gene. These variants may impact TM functions and compromise AqH drainage thereby contributing to elevated IOP and glaucoma.

## Results

### Identification of GLIS1 physiological functions

To obtain insights into the physiological functions of GLIS1^29,30^, *Glis1*-KO mice were examined for any potential phenotypic alterations. In these mice, most of exon 4 (840 bp) was replaced by lacZ containing three Stop codons (lacZ-Stop_3_) generating a fusion protein (GLIS1N-βGal) consisting of the N-terminus of GLIS1 and β-galactosidase (β-Gal). This protein lacks the entire DNA-binding domain (DBD) and C-terminus of GLIS1, including its transactivation domain (TAD) (Supplementary Fig. 1a). The fusion protein was undetectable by immunohistochemical staining for β-Gal (1:1000, PR-Z3781, Promega) in several tissues suggesting that it may be proteolytically degraded. Reporter transactivation analysis demonstrated that mutations in the ZF motifs that abolish their tetrahedral configuration, and deletion of the C-terminal TAD greatly decreased or fully abolished GLIS1 transcriptional activity (Supplementary Fig. 1b). These data indicate that loss of the ZFs and TAD in *Glis1*-KO mice abolishes the ability of GLIS1 to recognize the GLIS binding site (GLISBS) and to regulate the transcription of target genes. Supporting the specificity of the Glis1-KO, deletion of exon 4 had no significant effect on the expression of *Dmrtb1, Slc1a7, Dio1*, and *Cpt2*, genes neighboring *Glis1*, nor the expression of *Glis2* and *Glis3* in *Glis1*-KO kidneys and testes (Supplementary Fig. 1c, d).

**Fig. 1:**
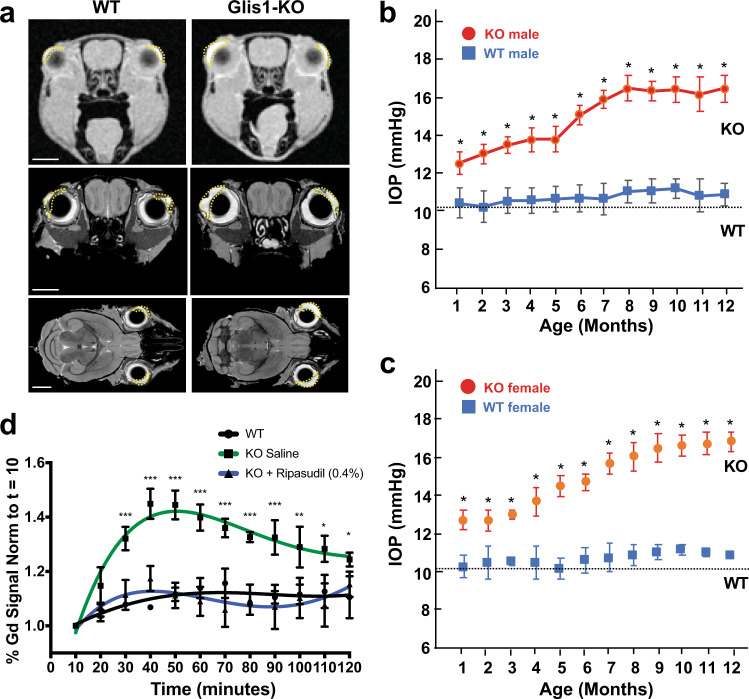
Anterior chamber is enlarged, IOP elevated, and AqH dynamics altered in *Glis1*-KO mice. **a** Representative MRI images from 2.5-month-old WT and *Glis1*-KO mice showing the increased size of the anterior chamber in *Glis1*-KO mice compared to WT. The upper two images are in vivo images acquired by dynamic contrast enhancement in the eye. The lower four images are coronal and sagittal sections of eyes from fixed specimen stained with gadolinium using active staining. The anterior chamber is outlined by the dotted line. Scale bar = 2 mm. **b**, **c** Comparison of IOP levels in male (**b**) and female (**c**) WT (squares) and Glis1-KO (circles) mice as a function of age. Male mice examined: at 1 and 2 months (*n* = 3); 3 (*n* = 5); 4 and 7 (*n* = 8); 5 (*n* = 6); 6, 11, and 12 months (*n* = 7). For female mice: 1 and 2 months (*n* = 10); 3–7, 9, and 10 months (*n* = 4); 8 (*n* = 5); 11 and 12 months (*n* = 3). IOP data from left and right eyes were combined and 4 IOP measurements/eye/time points were performed; thus, a total of 24–80 measurements at each time point. Data are represented as means ± SD. Statistical analyses were performed with a two-tailed Student’s *t* test. *p < 10^−5^. The dotted line indicates basal IOP level in 1–3-month-old mice. **d** AqH dynamics was examined by Gd-enhanced MRI over a 2 h period. Percent of Gd signal enhancement was determined and plotted. Left eye of 2-month-old WT mice (*n* = 3; black line) and *Glis1*-KO mice (*n* = 4; red line) was treated topically with saline and the right eye of Glis1-KO mice with Ripasudil (0.04%) (*n* = 4; blue line). Data are represented as means ± SD). Statistical analyses were performed with a two-tailed Student’s *t* test. *p < 10^−2^; **p < 10^−3^; ***p < 10^−4^.

Evaluation of 1–6 months C57BL/6NCrl *Glis1*-KO mice revealed that these mice developed enlarged eyes (Fig. 1a), while no other obvious abnormalities were observed. Similarly, no eye enlargement was observed in 129S6/SvEvTac *Glis1*-KO mice. Male and female KO mice in both backgrounds noticeably developed this abnormal eye phenotype between 2 and 3 months of age, which became more pronounced with age. Because protruding eyes are well-established comorbidity commonly associated with Graves’ disease, an autoimmune disease leading to hyperthyroidism^31^, and since GLIS1 and GLIS3 family members have been implicated in several thyroid gland-associated diseases^25,27,32,33^, we examined whether this *Glis1*-KO phenotype was related to the development of Graves’ disease that is characterized by high circulating levels of T3/T4 and low TSH. However, our analysis of serum T3, T4, and TSH showed that their levels were not significantly different between WT and *Glis1*-KO C57BL/6NCrl mice indicating that this phenotype was not related to the development of Graves’ disease (Supplementary Fig. 2).

### IOP is elevated in *Glis1*-KO mice

To examine whether the enlarged eye phenotype was associated with anatomic changes in intra- and periocular tissues, we performed Gadolinium magnetic resonance imaging (Gd-MRI) on the formalin-fixed specimens from 2.5-month-old WT and *Glis1*-KO C57BL/6NCrl mice. The contrast agent Gd has been used to assess ocular anatomy by MRI^34,35^. Analysis of multiple MRI images through the head revealed that there was little difference between the size of the periocular tissues in *Glis1*-KO relative to WT littermates in all three orientations. Importantly, we consistently observed an enlargement of the AC in both the right and left eyes of the *Glis1*-KO mice (Fig. 1a). The enlargement of AC observed in the *Glis1*-KO mice might be due to defective AqH drainage causing increased AqH accumulation that leads to the observed elevated IOP.

To obtain further support for this hypothesis, we measured IOP in WT and *Glis1*-KO mice over a 12-month time period. Our data demonstrated that IOP is significantly elevated in male as well as female *Glis1*-KO mice relative to age and gender-matched WT littermates (Fig. 1b, c). An increase in IOP was observed as early as in 1-month-old mice and then steadily increased before plateauing at 8 months. Further analysis revealed that the progressive, age-dependent increase in IOP was similar between left and right eyes (Supplementary Fig. 3). Together, these observations suggested a role for GLIS1 in the regulation of IOP.

### Decreased AqH outflow in eyes from GLIS1-deficient mice

Elevated IOP is most commonly caused by outflow resistance. To determine whether the elevated IOP in *Glis1*-KO C57BL/6NCrl mice was due to changes in AqH drainage, we employed dynamic contrast-enhanced MRI to evaluate AqH dynamics in vivo. The contrast agent Gd present in the AC enhances the T1-weighted MRI signal brightness and serves as a tracer, thereby providing a readout for AqH accumulation and outflow^34,35^. Following administration of Gd, 2 months-old mice were scanned for 2 h at 10 min intervals and Gd accumulation in the AC was measured relative to the initial baseline (see image source files at https://civmvoxport.vm.duke.edu/voxbase/studyhome.php?studyid=733). Our data indicated that in WT mice Gd is readily cleared from the eye (Fig. 1d). A significant (29%; *p* < 0.0001) increase in Gd accumulation in the AC was detected in *Glis1*-KO eyes as compared to the WT eyes suggesting impaired AqH exit. It is well-established that a major route of AqH exit from the AC is via the conventional drainage pathway comprising the TM and SC^10^. To obtain further support for this hypothesis, we evaluated the AqH dynamics in *Glis1*-KO mice following topical administration (5 μl, 0.4%) of Ripasudil. This drug functions as an IOP lowering Rho-kinase inhibitor that enhances AqH outflow via the TM and SC^36^. As shown in Fig. 1d, Ripasudil treatment significantly reduced Gd accumulation in the ocular AC of *Glis1*-KO mice as compared to treatment with isotonic saline in the contralateral eye consistent with its IOP lowering effects. Our data suggest that the increased IOP observed in the *Glis1*-KO mice correlates with reduced AqH outflow and might involve dysfunction of the TM, a major cause of elevated IOP and glaucoma.

### Progressive disruption of ocular drainage structures in *Glis1*-KO mice

**Fig. 2:**
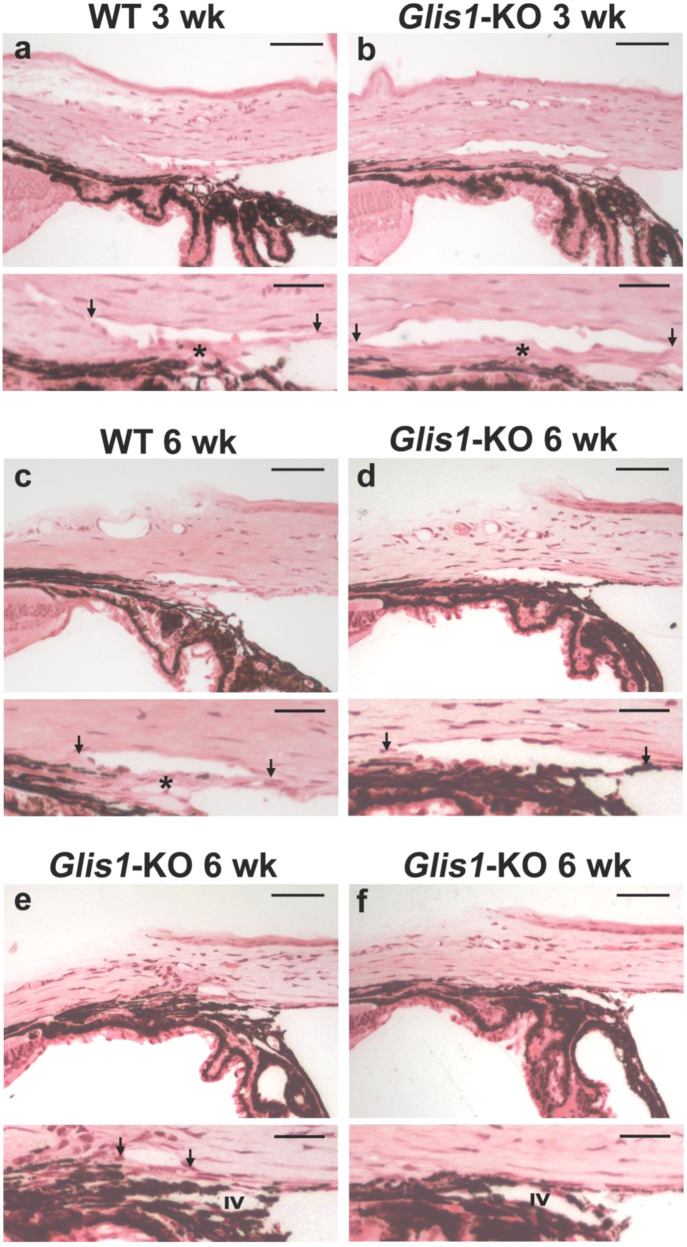
Disruption of the ocular angle drainage structures in *Glis1*-KO mice. **a**, **c** WT mice maintained in C57BL/6NCrl background showed a well-developed SC and TM (*) at both 3 and 6 weeks of age. **b** *Glis1*-KO-C57BL/6NCrl eyes exhibit a morphologically mature ocular drainage tissue at 3 weeks of age. The histological assessment of the ocular angle at 3 weeks of age was performed on 6 WT and 6 Glis1-KO eyes with similar results within each group. In contrast, by 6 weeks of age *Glis1*-KO eyes exhibit a variable degree of focal TM degeneration, ranging from hypoplastic TM characterized by substantial thinning of the TM (**d**), reduction in the size of the Schlemm’s canal, and associated TM causing a partial collapse of the ocular drainage structures (**e**)**. f** At 6 weeks of age a small proportion of mice (<10%) showed focal regions exhibiting partial or complete collapse of the ocular drainage structures lacking TM and Schlemm’s canal. **a**–**f** A magnified version of the image in the upper panel is shown in the lower panel. Arrows show edges of the SC. IV Iris vessel. The histological assessment of the ocular angle at 6 weeks of age was performed on 10 WT and 15 Glis1-KO eyes with similar results within each group. Scale bar = 50 μm. Detailed measurement of the TM area was performed on 5 eyes per experimental group, shown in Supplementary Figs. 5 and 6.

To determine whether structural and morphological changes of ocular drainage tissues in *Glis1*-KO mice might underlie TM dysfunction and elevated IOP, we performed a detailed ocular histological examination of WT, *Glis1*-heterozygous and *Glis1*-KO mice maintained in a C57BL/6NCrl strain. Ocular angle structures of the *Glis1*-heterozygous mice showed an intact ocular drainage tissue, unlike the *Glis1*-KO that exhibited TM degeneration (Supplementary Fig. 4a, b). This lack of phenotype in heterozygous mice is consistent with that these mice did not develop elevated IOP (Supplementary Fig. 4c). Histological analyses demonstrated that the angle structures of the *Glis1*-KO eyes initially appear normal. At 3 weeks, no significant difference in TM morphology was observed suggesting that GLIS1 has no major effect on TM development (Fig. 2a, b; Supplementary Fig. 5). Major phenotypic changes are observed by 6–8 weeks of age (Fig. 2c–f). Focal regions of the angles in 6-week-old *Glis1*-KO mice exhibited thinning of the TM (hypoplasia) in a substantial proportion of mutant eyes (Fig. 2d; Supplementary Fig. 6), while some local regions lacked discernible TM. Based on histology, the damage to the ocular drainage structure within an eye is quite variable at earlier time points (6 weeks) with some regions appearing much more normal. Such local variability is well documented for other glaucoma genes and may partially explain the relatively modest increase in IOP^37,38^.

The loss of GLIS1 function did not affect the gross morphology of the SC at an early time point (4 weeks) (Supplementary Fig. 7a, b) when the TM is still largely intact. However, at later ages (6 weeks and older), but more common at 3 months and older ages, there are regions where the SC becomes partially or completely collapsed (Fig. 2e, f). This might be due to a regional or complete degeneration of the TM that may protect the SC from collapse. At older ages (over 6 months), in addition to the degeneration of the TM and collapse of the ocular drainage structures, *Glis1*-KO eyes exhibited anterior synechiae characterized by a fusion of the iris and cornea causing angle closure (Supplementary Fig. 8a). Besides the observed defects in the ocular drainage structures, no gross abnormalities were observed in other ocular tissues (Supplementary Figs. 7c and 8b). We also characterized the ocular angle of *Glis1*-KO mice maintained in a 129S6/SvEvTac background. These mice exhibited thinning of the TM layer like that observed in the C57BL/6NCrl background (Supplementary Fig. 9). Our data suggest that GLIS1 deficiency leads to progressive TM dysfunction and TM degeneration.

### GLIS1 is highly expressed in TM cells

**Fig. 3:**
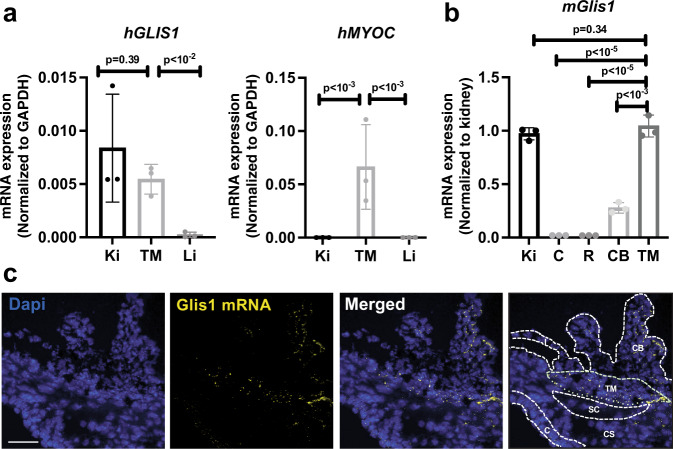
GLIS1 is highly expressed in TM. **a** QPCR analysis of GLIS1 and MYOC mRNA expression in human TM tissue, kidney (Ki), and liver (Li) (*n* = 3; technical replicates). Statistical analyses were performed with a two-tailed Student’s *t* test. Data are represented as means ± SD. *p* Values are indicated above the bars. **b** Comparison of mouse GLIS1 RNA expression in several ocular tissues with that of kidney, a tissue in which GLIS1 is highly expressed (*n* = 3; distinct samples). Statistical analyses were done with a two-tailed Student’s *t* test. Data are represented as means ± SD. *p* Values are indicated above the bars. **c** RNAscope in situ hybridization with eye sections from a 3-month-old WT mouse showed that GLIS1 mRNA (yellow speckles) expression was restricted to TM and CB. Dashed lines outline different cell compartments. Kidney (Ki), Cornea (C), Retina (R), Ciliary Body (CB), Trabecular Meshwork (TM), Corneal StromaI (CS), Iris (I), and Schlemm’s canal (SC). Scale bar = 50 μm.

Since the TM plays a major role in the regulation of AqH drainage and IOP, we decided to focus our study on the analysis of the TM. To examine whether the observed changes in the TM might be intrinsic to the loss of *GLIS1* expression in this tissue, we analyzed *GLIS1* expression in human TM (HTM) tissue and primary HTM cells. The human *GLIS1* gene and its mouse orthologue can generate two transcripts, long and short (referred to as GLIS1_L_ and GLIS1_S_) that generate a 795 or a 620 amino acids protein, respectively (a 789 and 620 amino acid protein in mice) (https://useast.ensembl.org/Homo_sapiens/Gene/Summary?db=core;g=ENSG00000174332;r=1:53506237-53738106). QPCR analysis demonstrated that GLIS1_L_ was the primary transcript in primary HTM cells, and all human tissues tested (Supplementary Fig. 10), whereas GLIS1_S_ was expressed at very low levels. In isolated HTM, characterized by their high myocilin (MYOC) expression, GLIS1 mRNA was expressed at levels comparable to that of the kidney, a tissue in which GLIS1 is highly expressed^29^ (Fig. 3a). qPCR analysis further showed that GLIS1 mRNA was highly expressed in mouse ocular tissue enriched in the TM, moderately in the ciliary body, and at very low levels in the cornea and retina (Fig. 3b). In situ RNA localization by RNAscope supported the expression of Glis1 transcripts in the TM and ciliary body isolated from 3-month-old WT mice (Fig. 3c), whereas Glis1 transcripts were not detectable in the iris and cornea. These data indicated that *GLIS1* expression is intrinsic to TM cells and suggests that the TM dysfunction observed in *Glis1*-KO mice is likely causally related to the loss of GLIS1 transcription activation function in these cells. In contrast to TM, the ciliary body, which also expressed Glis1, exhibited a properly organized epithelial layer (Supplementary Fig. 7c).

### Regulation of gene expression by GLIS1 in primary human TM cells

**Fig. 4:**
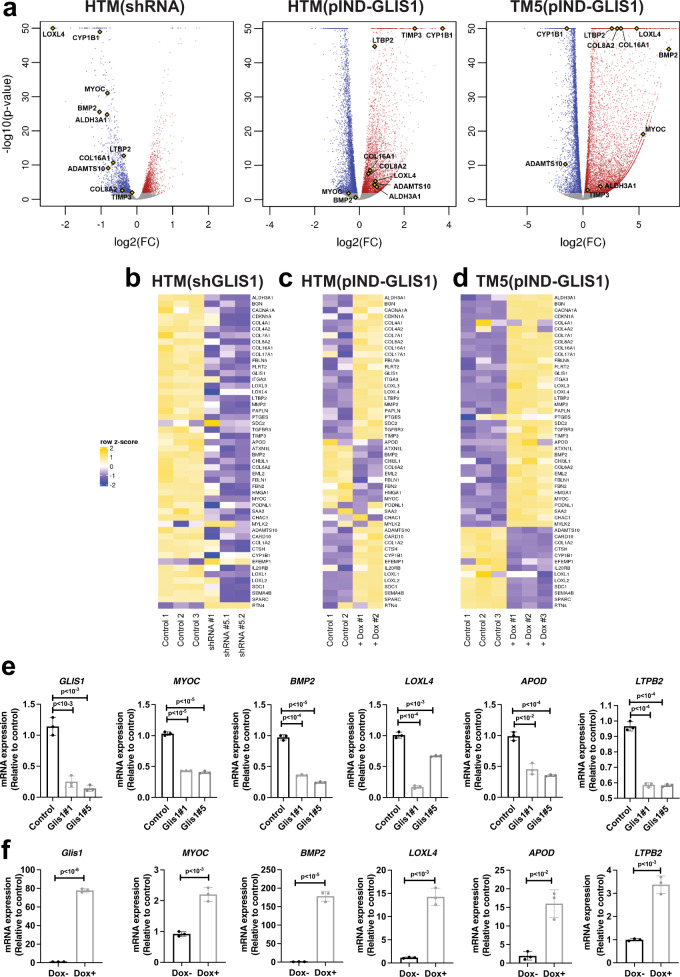
Regulation of TM/glaucoma-related gene expression by GLIS1 in TM cells. **a** Volcano plots of genes downregulated (blue) and upregulated (red) in HTM(shGLIS1) and Dox-treated HTM(pIND-GLIS1) and TM5(pIND-GLIS1) cells (as determined by DESeq2 at FDR 0.01). All other genes are in gray. Several genes associated with IOP, glaucoma, or ECM are indicated (yellow diamonds). The *x*-axis represents gene expression log2-fold change (FC) and the *y*-axis represents −log10 (*p*-value). **b** Heatmap of the differential expression of several TM-, glaucoma-, ECM-related mRNAs in HTM(shGLIS1), and HTM(Scr) (Control) cells; underlying data are rlog-transformed quantification scores as reported by DESeq2 followed by row-scaling at FDR 0.01. Data shown are for HTM (Scr), shGLIS1#1, and shGLIS1#5 replicates 1 and 2. **c**, **d** Heatmap of the differential expression of several TM-, glaucoma-, ECM-related mRNAs in HTM(pIND-GLIS1) cells (**c**) expressing Dox-inducible Flag‐GLIS1‐HA treated for 18 h with or without Dox (Control) (*n* = 2) and TM5(pIND-GLIS1) cells (*n* = 3) (**d**). **e** qPCR analysis of several genes downregulated in HTM(shGLIS1) compared to HTM(Scr) cells (*n* = 3, independent replicates). **f** qPCR analysis (*n* = 3, independent replicates) of several genes induced by murine GLIS1 in TM5 cells expressing Dox-inducible Flag‐GLIS1‐HA. Cells were treated for 18 h with or without Dox (±Dox). Data in **e** and **f** are represented as means ± SD. Statistical analyses were performed with a two-tailed Student’s *t* test. *p* Values are shown above the bars.

GLIS1 regulates gene transcription by binding to GLISBS in the promoter regulatory region of target genes^25,26^. To investigate alterations in gene expression that might underlie the phenotypic changes observed in TM cells, we performed RNA-Seq and ChIP-Seq analyses. Transcriptome analysis was performed with HTM(shGLIS1) cells, in which GLIS1 expression was knocked down by GLIS1 shRNA lentivirus, and with control cells (HTM (Scr)) infected with scrambled shRNA lentivirus. The volcano plot in Fig. 4a shows the distribution of downregulated and upregulated genes in HTM(shGLIS1) cells in comparison to HTM(Scr) cells. In addition to downregulation of GLIS1 mRNA, the expression of several genes associated with TM functions was decreased in HTM(shGLIS1) cells, including *MYOC*, *CHI3L1*, *SPARC*, *CYP1B1*, and *APOD*^39–41^(Fig. 4a, b; Supplementary Table 2). In addition, the expression of a variety of genes encoding extracellular matrix (ECM) components was reduced in HTM(shGLIS1) cells, including a number of collagen genes (e.g., *COL1A2*, *COL6A2*, and *COL4A1/2*), fibulins (*FBLN1* and *FBLN5*), microfibril-related genes (*FBN2*, *LOXL1-4*, and *LTBP2*), matrix metalloproteinases (*ADAMTS10* and *MMP2*), and genes involved in cell-cell and cell-ECM adhesion (e.g., *ITGA3*) (Fig. 4a, b; Supplementary Table 2)^42^. In addition, the expression of a number of genes was up-regulated, including *EFEMP1* and *RTN4*. qPCR-analysis confirmed the decrease in MYOC, BMP2, LOXL4, APOD, LTBP2, and CYP1B1 mRNA expression in HTM(shGLIS1) cells (Fig. 4e and Supplementary Fig. 11). Several of the differentially expressed genes have previously been reported to be associated with elevated IOP and/or glaucoma, including *MYOC*, *ADAMTS10*, *LTBP2*, *LOXL1*, *TGFBR3*, *CYP1B1*, and *EFEMP1*^5,28,43–45^.

The regulation of many of these genes by GLIS1 was further supported by gene expression analysis in TM cells overexpressing GLIS1. For this analysis we used HTM(pIND-GLIS1) transiently expressing Dox-inducible Flag-GLIS1-HA and TM5(pIND-GLIS1), stably expressing a Dox-inducible Flag-GLIS1-HA. Dox treatment greatly induced GLIS1 mRNA expression in TM5(pIND-GLIS1) cells and accumulation of Flag-GLIS1-HA protein in the nucleus (Fig. 4f; Supplementary Figure 12). Transcriptome analysis showed that induction of GLIS1 expression in Dox-treated HTM(pIND-GLIS1) and TM5(pIND-GLIS1) cells enhanced the expression of many, but not all, of the same genes that were down-regulated by shGLIS1 RNAs in HTM cells (Fig. 4a, c, d; Supplementary Table 2). QRT-PCR analysis showed that the decreased expression of *MYOC*, *BMP2*, *LOXL4*, *APOD*, and *LTBP2* in HTM(shGLIS1) correlated with increased expression in Dox-treated TM5(pIND-GLIS1) cells (Fig. 4e, f). Similarly, the induction of CYP1B1 mRNA in HTM(pIND-GLIS1) cells correlated with decreased expression in HTM(shGLIS1) (Supplementary Fig. 11). As indicated above, decreased expression in HTM(shGLIS1) did not always perfectly correlate with increased expression in HTM(pIND-GLIS1) and/or TM5(pIND-GLIS1) cells (Supplementary Table 2). Such differences might, among other things, be due to variations in the epigenome and the transcription regulatory machinery between primary and immortalized TM cells or different efficiencies of the shGLIS1 used. It might further relate to differences in the expression levels of endogenous GLIS1 or that of GLIS1 target genes in HTM vs. TM5 cells or variations in the binding affinity of GLIS1 to GLISBS of target genes.

**Fig. 5:**
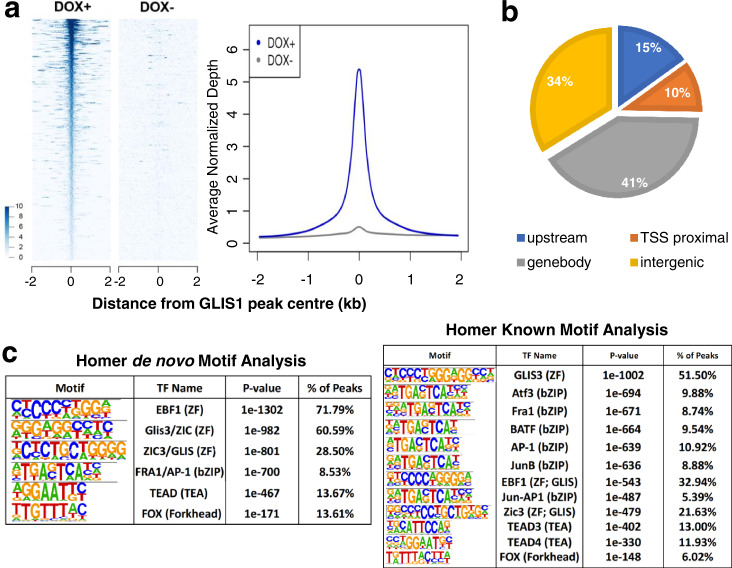
GLIS1 regulates the transcription of a subset of TM/glaucoma-related genes in HTM cells through its interaction with GLISBS. **a** Heatmap and ChIP-Seq read density plot showing GLIS1 occupancy in TM5(pIND-GLIS1) cells treated for 18 h with Dox (+Dox) compared to untreated cells (−Dox). Each line in the heatmap represents an individual GLIS1 binding site. **b** Pie chart showing the location of GLIS3-binding peaks within specific regions of the genome. TSS proximal: −1 kb to the transcription start site (TSS); upstream: −1 to −10 kb. **c** Homer de novo and known motif analysis identified GLIS-like binding sites (GLISBS) as the top consensus sequence motif. Binding sites for transcription factors of the AP-1, FOX, and TEAD families were identified alongside GLIS consensus motifs.

To determine which of the differentially expressed genes were direct transcriptional targets (cistrome) of GLIS1, we performed a ChIP-Seq analysis. Since no suitable GLIS1 antibody was available for ChIP-Seq and it was not feasible to establish primary HTM cells stably expressing GLIS1, hence we utilized the TM5(pIND-GLIS1) cells. ChIP-Seq analysis to identify direct transcriptional targets of GLIS1 showed enrichment for GLIS1 binding (Fig. 5a). ChIP-Seq analysis identified a total of 46,947 distinct GLIS1 binding peaks in Dox-treated TM5(pIND-GLIS1) cells. About 10% of GLIS1 binding peaks were within proximal promoter regions 1 kb upstream of the transcription start sites (TSSs), whereas 16% were further upstream (Fig. 5b). GLIS1 binding was most highly enriched at introns within the gene body as we reported for GLIS3^32,46^. Homer de novo and known motif analyses identified a G/C-rich GLISBS-like consensus sequence as the top motifs (Fig. 5c). This sequence was very similar to the consensus GLISBS reported previously^32,46,47^, indicating that our ChIP-Seq was successful in detecting specific GLIS1 binding sequences. Our GLIS1 ChIP-Seq analysis identified a number of additional motifs, including motifs for bZIP transcription factors (e.g., ATF3, FRA1, BATF, and JUNB, members of the AP-1 complex), forkhead box (FOX) proteins^48^, and TEA domain transcription factors (TEAD) that play a key role in the Hippo pathway^49^. These data suggested co-localization of the GLIS1 binding consensus with motifs for other transcription factors that have been previously implicated in the regulation of TM and glaucoma^45,50,51^. These findings are consistent with the hypothesis that GLIS1 regulates TM gene transcription in coordination with other transcription factors.

**Fig. 6:**
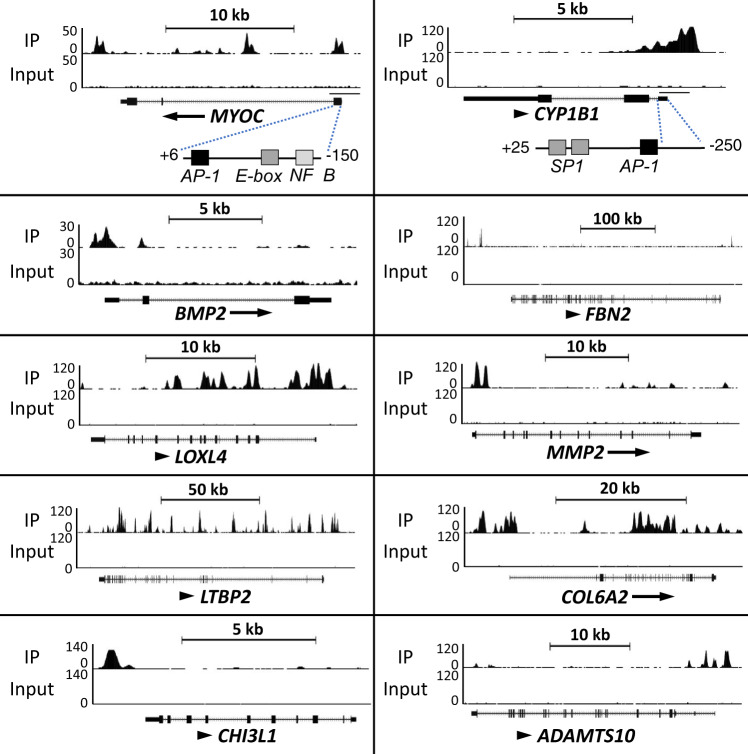
Genome browser tracks of the MYOC, CHI3L1, BMP2, FBN2, LOXL4, MMP2, LTBP2, COL6A2, CYP1B1, and ADAMTS10 genes (https://genome.ucsc.edu/) showing GLIS1 occupancy (ChIP-Seq) in TM5(pIND-GLIS1) cells expressing Flag-GLIS1-HA. The AP-1, E-box, and NFκB binding sites in *MYOC* and the AP-1 and G/C-rich SP1-binding sites in the *CYP1B1* proximal promoter region are indicated.

Analysis of the combined RNA-Seq and ChIP-Seq data showed that the transcription of several of the differentially expressed genes with roles in TM, IOP, and glaucoma, were directly regulated by GLIS1 and included *MYOC*, *LTBP2*, *CHI3L1*, *HMGA1*, *CYP1B1*, and *LOXL1-4* (Supplementary Table 2). Genome browser tracks showing the location of GLIS1 peaks associated with several glaucoma-related genes, including *MYOC*, *CYP1B1*, and *ADAMTS10*, are shown in Fig. 6. Interestingly, the GLIS1 binding peaks in the proximal promoter regions of *MYOC* and *CYP1B1* are located in a region near a functional AP-1 responsive element (Figs. 5c and 6)^52–54^. In addition, the proximal promoter of CYP1B1 contains a G/C-rich SP1 binding sequence, which because of its similarity to the consensus GLISBS might function as a GLIS1 binding site. This suggests that these promoter regions may function as a regulatory hub for several transcription factors. Moreover, this supports our hypothesis that the transcription of *MYOC*, *CYP1B1*, and other TM genes by GLIS1 is regulated in coordination with other transcription factors, including members of the AP-1 family.

KEGG pathways analysis of GLIS1 target genes down-regulated in HTM(shGLIS1), activated in HTM(pIND-GLIS1) or TM5(pIND-GLIS1) cells identified pathways associated with ECM, proteoglycans, and cellular adhesion among the top pathways in all three data sets (Supplementary Fig. 13). This is consistent with recent bioinformatics analyses of TM gene expression data that identified cell–matrix and cell–cell interaction-related pathways among the top pathways involved in the pathogenesis of POAG^55^.

### Association of *GLIS1* rs941125 with glaucoma

**Fig. 7:**
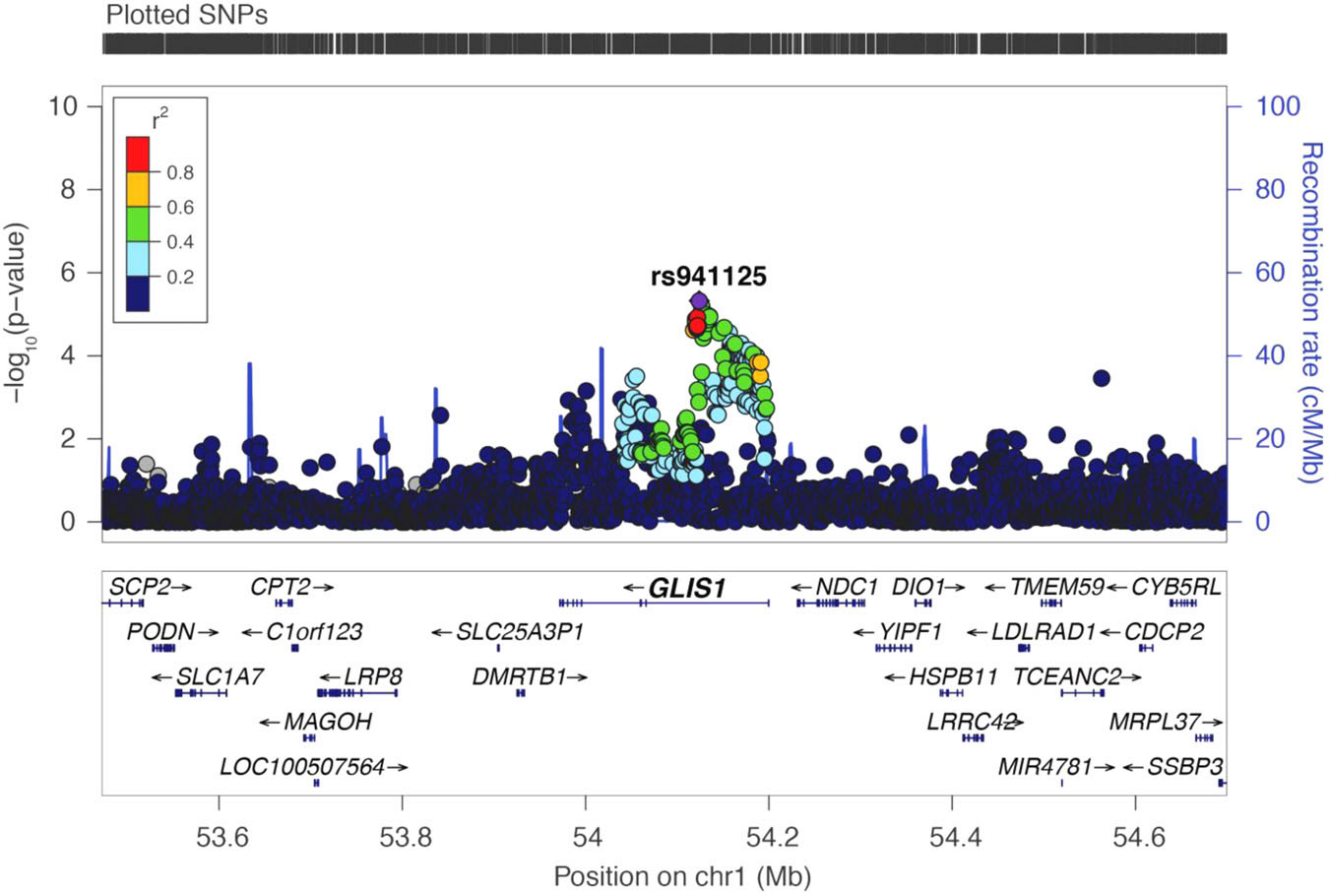
Regional plot at the GLIS1 genomic region showing association with glaucoma in the combined (GERA + UK Biobank) multiethnic meta-analysis. Top SNP rs941125 is significantly associated with glaucoma after Bonferroni correction (*p* = 4.73 × 10^−6^).

Our study of *Glis1*-KO mice identified a critical regulatory function for GLIS1 in the maintenance and function of the TM, a tissue that plays a major role in controlling AqH outflow and the development of glaucoma^1,15,56,57^. These findings raised the question of whether *GLIS1* might be involved in the pathogenesis of human glaucoma as well. To assess this, we examined the association of SNPs in the *GLIS1* region and the risk of glaucoma, combining information from the GERA and UKB cohorts^9^. rs941125, which localizes to intron 1 of *GLIS1*, was the most strongly associated SNP in the region, reaching a Bonferroni corrected level of significance (odds ratio = 0.94, *p* = 4.73 × 10^−6^) (Fig. 7 and Supplementary Data 1). The association of rs941125 with POAG has recently been confirmed and replicated in additional cohorts at a genome-wide level of significance (*p* = 2.01 × 10^−11^, meta-analysis)^58^. Furthermore, rs941125 is significantly associated with variation in *GLIS1* gene expression in several tissues in GTEx (https://gtexportal.org/home/snp/rs941125). Together, these findings support a role for *GLIS1* in glaucoma pathogenesis in humans.

## Discussion

In this study, we identify a critical role for the transcription factor GLIS1 in the maintenance of TM/ocular drainage tissue, AqH dynamics, and IOP. The TM is an essential component of the ocular drainage structure and TM abnormalities play a major role in the development of elevated IOP and glaucoma^1,5,15,57,59,60^. Utilizing *Glis1*-KO mice, we demonstrate that the loss of GLIS1 function causes a progressive degeneration of the TM leading to a disruption of ocular drainage structures. As a consequence of these changes, the AqH drainage is significantly compromised in *Glis1*-KO eyes causing elevated IOP. In addition, we identified several transcriptional targets of GLIS1 in TM cells that previously have been implicated in TM-related functions, IOP homeostasis, and ocular hypertension/glaucoma, including *MYOC*, *ADAMTS10*, *LTBP2*, *LOXL1*, *TGFBR3*, *CYP1B1*, and *EFEMP1*^5,28,43,44^. The reduced expression of a set of TM genes together is likely responsible for the TM dysfunction and elevated IOP in Glis1-KO mice. Importantly, we have detected significant associations between common genetic variants in the GLIS1 region and POAG in humans, thereby supporting the role of GLIS1 as a glaucoma risk gene. These variants may impact TM functions and compromise AqH drainage by altering GLIS1 expression and/or function and leading to elevated IOP and glaucoma.

The expression of *Glis1* in the TM and the degeneration of the TM in *Glis1*-KO mice suggested that GLIS1 plays a critical regulatory role in maintaining TM cell function and survival. It is possible that GLIS1 deficiency leads to the disruption of a cell-intrinsic biological process important in maintaining normal structure and TM function. Excessive loss of TM cells has been proposed to be a critical pathophysiological feature resulting in defective AqH drainage and high IOP^14^. Although the pronounced loss of TM cells in POAG patients was observed decades ago^13,61^, much remains yet to be understood with regards to cellular processes regulating the maintenance of the TM and survival of TM cells and its relevance to IOP and the different forms of glaucoma^14,15,56^. Various changes in the TM, including alterations in ECM and mitochondria, increased oxidative stress, and apoptosis, have been implicated in outflow resistance, elevated IOP, and increased glaucoma risk^15,41,42,55,62–65^. Specifically, the ECM has been identified as a major player in maintaining the structural integrity and functionality of the TM^55,59,61,64–66^. Consistent with this, our gene profiling analyses identified ECM-related processes, cytoskeleton, and cellular adhesion as key pathways that are impacted as a consequence of *GLIS1* knockdown or ectopic expression of *GLIS1* (Fig. 4e, f; Supplementary Fig. 13). Moreover, several ECM-related genes, which levels changed in TM cells upon GLIS1 knockdown or overexpression, have previously been implicated in elevated IOP and glaucoma, including members of the collagen I, IV, and VI families, *LTBP2*, a regulator of TGFβ signaling and ECM deposition^28,67^. Furthermore, the expression of a number of microfibril-associated genes is also impacted, including FBN2, encoding a microfibril-associated glycoprotein that contributes to elastin assembly in the ECM of the TM^60,68^. It further includes *LOXL1–4*, encoding lysyl oxidases that mediate the cross-linking of several ECM proteins, such as collagens and elastin^21,69^, and *ADAMTS10*, encoding a metalloproteinase involved in ECM assembly^44,70^. Alterations in the expression of these genes are likely to impact ECM assembly as well as its biomechanical properties, and physiological processes, such as differentiation, survival, and tissue organization that are regulated by the ECM-dependent signaling pathways^71^. Thus, altered cell-ECM interaction or abnormal ECM organization in *Glis1*-KO mice might adversely affect the maintenance and biomechanical properties of the TM, thereby leading to progressive degeneration of the TM and disruption of the AqH drainage and subsequently to the development of elevated IOP and glaucoma^14,42,62,63^. Given a role for ECM-dependent pathways, it is possible that change in biomechanical properties of the TM (TM stiffness) may contribute to IOP changes, especially during an early time window prior to any obvious structural changes not captured by histological assessment.

In addition to these ECM and adhesion-related genes, transcriptome analysis showed that GLIS1 impacts the expression of several other TM-, IOP-, and glaucoma-related genes, including *MYOC* and *CYP1B1* (Fig. 4a, e, f; Supplementary Fig. 12). It is interesting to note that the phenotypic changes in the TM observed in *Glis1*-KO mice exhibit some resemblance with those seen in *Cyp1b1*-deficient mice, including the collapse and degeneration of the TM^37,61^. Thus, the reduced *CYP1B1* expression might contribute to the structural changes in the TM and elevated IOP observed in *Glis1*-KO mice. In the case of MYOC, mutations in *MYOC* are thought to act by a gain of function mechanism resulting in the misfolding and accumulation of mutant MYOC leading to ER stress and apoptosis of TM cells^72,73^. Moreover, studies with *Myoc* knockout mice suggest that loss of *Myoc* function by itself does not cause ocular drainage tissue abnormalities or IOP elevation^74^. These studies indicate that reduced expression of *M*yoc observed in Glis1-KO mice is unlikely by itself inducing TM abnormalities and elevated IOP. We hypothesize that reduced expression of a set of genes rather than one particular single gene is causing the TM abnormalities and high IOP in GLIS1 deficiency, as we reported for the development of congenital hypothyroidism and neonatal diabetes in Glis3-KO mice32,47. Thus, the altered expression of a set of TM genes, including CYP1B1, ADAMTS10, and LTBP2, may underlie TM dysfunction and elevated IOP. Therefore, the effect of Glis1 deficiency on mouse TM is likely a cumulative effect of disruption of multiple pathways.

To establish which of the differentially expressed genes were directly regulated by GLIS1, we performed a ChIP-Seq analysis. This analysis revealed that GLIS1 binding was associated with *MYOC*, *ADAMTS10*, *CYP1B1*, *MMP2*, and many other genes, suggesting that these genes are direct transcriptional targets of GLIS1 (Supplementary Table 2). Interestingly, many of the direct targets of GLIS1 have a role in the ECM or TM function and have been implicated in the pathogenesis of elevated IOP and glaucoma^6,28,44,56^. Homer motif analysis suggested co-localization of GLIS1 binding peaks with motifs of other transcription factors, including binding sites for members of the AP-1, TEAD, and forkhead box (FOX) families (Fig. 5). Interestingly, the proximal promoters of *MYOC* and *CYP1B1* have been reported to contain functional binding sites for AP1-related transcription factors near the location of the GLIS1 binding peaks (Fig. 6)^52–54,75,76^. The forkhead box member, FOXC1, has been implicated in the regulation of TM functions and glaucoma^77,78^, while the Hippo pathway through activation of TEAD transcription factors regulates ECM in TM cells and appears to have a role in glaucoma^50,51,79^. Together, these observations support a model in which a selective set of GLIS1 target genes are co-regulated with other transcription factors through their interaction within the same regulatory regions in TM-specific genes.

Our genetic studies provide further validation for the role of GLIS1 in glaucoma pathogenesis and extend our findings in mice to humans. The observed ocular drainage defects exhibited by *Glis1*-KO mice are more severe than those observed in POAG and likely due to a complete deficiency of GLIS1 and the suppression of many target genes, as compared to TM-specific changes originating from potential gene dosage effects of POAG-associated variants. The lead SNP rs941125 is not only associated with glaucoma in multiple independent cohorts^58^, it is also detected as an eQTL associated with a change in *GLIS1* expression (https://gtexportal.org/home/snp/rs941125). We note, however, that GTEx does not contain information on eye tissues and that the eQTL analysis for rs941125 is based on expression data from brain tissue. Future studies need to determine whether rs941125 or other SNPs in linkage disequilibrium with rs941125 are located within gene regulatory elements (such as enhancers) that affect *GLIS1* expression and how alteration of these regulatory elements predispose the eyes to TM dysfunction leading to IOP elevation. Since POAG is multifactorial, it is likely that variants in other genes together with *GLIS1* SNPs may cooperate to induce high IOP in patients. The absence of such other modifier alleles in *Glis1* heterozygous mice might explain why the presence of a single knockout allele of *Glis1* is not sufficient to induce ocular drainage defects leading to elevated IOP.

In this study, we identify a critical role for GLIS1 in the maintenance and regulation of TM function, AqH dynamics, and IOP. GLIS1 together with other transcription factors might be part of a regulatory network required for proper maintenance and functioning of ocular drainage tissue and IOP homeostasis^45^. Thus, *Glis1-*KO mice provide us with a valuable model to uncover cellular and molecular mechanisms that underlie the regulation of TM maintenance and ocular drainage tissue homeostasis and potentially lead to new insights into the pathogenesis of glaucoma. In addition, our data further suggest that altered expression of *GLIS1* in individuals carrying the risk allele may confer increased susceptibility toward developing POAG possibly by impacting the TM and thereby contributing to elevated IOP. Finally, as has been shown for the hedgehog/GLI signaling pathway, regulation of GLIS proteins^25^ by primary cilium-associated G protein-coupled receptors might be useful for the development of new therapeutic strategies in the management of various pathologies, including glaucoma.

## Methods

### Glis1-deficient mice

Glis1-deficient mice (*Glis1*-KO) were described previously^30^. Mice were bred into the C57BL/6NCrl Charles River, Wilmington, MA) and 129S6/SvEvTac (Taconic, Rensselaer, NY) backgrounds for at least seven generations. We ensured that mice maintained in C57BL/6NCrl neither carried homozygous RD8 mutation nor exhibited retinal degeneration (based on an ocular histological assessment). *Glis1*-KO mice on both backgrounds developed enlarged eyes and appeared to exhibit a similar phenotype. Most experiments were carried out with *Glis1*-KO C57BL/6NCrl mice. Mice were supplied ad libitum with autoclaved NIH-31 rodent diet (Harlan Laboratories, Madison, WI) and provided with distilled drinking water and were group-housed in individually ventilated cages (Techniplast, Exton, PA). Experiments took place in an AAALAC accredited facility maintained at 70–73 °F, relative humidity 40–60%, and 12 h:12 h light–dark cycle. All mice were negative for rodent murine pathogens. Littermate wild-type (WT) mice were used as controls. All animal protocols followed the guidelines outlined by the NIH Guide for the Care and Use of Laboratory Animals and were approved by the Institutional Animal Care and Use Committee at the NIEHS. Routine genotyping was carried out with the following primers: Glis1-F, 5′-AGCTAGTGGCTTTCGCCAACA; Glis1-R, 5′-GAACAAGATAGAATCATGG-TATATCC and Neo-pro, 5′-ACGCGTCACCTTAATATGCG.

### T3, T4, and thyroid-stimulating hormone (TSH) assays

Blood levels of T3 and T4 were measured by radioimmunoassay (MP Biomedicals, Orangeburg, NY) as described previously^32^. Serum TSH was analyzed with a mouse pituitary magnetic bead panel kit (EMD Millipore Corp., Billerica, MA.

### RNAscope in situ hybridization

In situ hybridization was carried out on formalin-fixed paraffin-embedded tissue sections using the RNAScope system (Advanced Cell Diagnostic, Hayward, CA) per manufacturer’s instructions and as previously described^80^.

### IOP measurement

IOP of both eyes in age- and gender-matched WT and *Glis1*-KO littermates was measured using the Icare TonoLab rebound tonometer (Icare, Helsinki, Finland). Immediately prior to measurement animals were briefly sedated using isoflurane. IOP was measured at least 4 times/eye within a 30 min time frame with each individual IOP recorded representing the average of six measurements, giving a total of 24 rebounds from the same eye.

### Ocular angle assessment

Hematoxylin and eosin-stained ocular sections cut from plastic embedded eyes were assessed to examine the morphology of the ocular angle structures. Briefly, mice were euthanized and eyes enucleated and immediately immersed in cold fixative (1% PFA, 2% glutaraldehyde, and 0.1 M cacodylate buffer) for at least 48 h, after which they were transferred to cold 0.1 M cacodylate buffer solution and stored at 4 °C. We have found that using this fixative, greatly improves capturing the SC in its intact/non-collapsed conformation. For example, at around 3 months of age 100% of WT eyes showed intact SC. Samples were embedded in glycol methacrylate, and serial sagittal sections (2 μm) passing through the optic nerve were cut and stained with hematoxylin and eosin (H&E). Ten similarly spaced sections corresponding to the peripheral, mid-peripheral, and central regions (passing through the optic nerve) of the eye were evaluated^81^. Both angles of a section were considered for evaluation. The prominent angle-relevant structures, including TM, SC, Iris, ciliary processes were histologically examined. A representative image showing angle-relevant tissues is indicated in Supplementary Fig. 14. Eyes assessed for each of genotype included both sexes.

The sections corresponding to the central region of the eye were used for measuring the TM area, which in our experience provides the most reliable assessment. We imaged on average five consecutive serial sections per eye from both wild type and *Glis1-* KO mice. Cross-section images were taken using a 20× objective on the Zeiss Axiophot microscope with a 12 Mp Insight camera. TM images were captured by SPOT5.6 imaging software, assigned an accurate 20× calibration (to account for the magnification of the acquired image), and the TM area (marked in Supplementary Figs. 5 and 6) was measured using the “region” tool under the EDIT menu. The mean TM area was calculated from a minimum of five central ocular sections/eye.

### Assessment of the SC

Briefly, the anterior segment is excised and the iris removed. The anterior segment cup is relaxed by making four centripetal cuts. These cuts generated four fan-shaped quadrants attached at the center. The SC runs along the rim (limbus) of each fan-shaped quadrant. Whole mounts of the anterior segments from control and *Glis1*-KO mice were stained with an endomucin antibody^82^. Briefly, the anterior segment is excised and the iris removed. The anterior segment cup is relaxed by making four centripetal cuts. These cuts generated four fan-shaped quadrants attached at the center. The SC runs along the rim (limbus) of each fan-shaped quadrant. The anterior segment was stained with endomucin (5 µg/ml; Thermo Fisher Scientific), the whole mounted, and the entire limbus encompassing each of the four quadrants imaged using a Leica LSM SP8 confocal system and DM6000 vertical microscope. The limbal region was imaged with a 20×/0.75 IMM CORR CS2-multi-immersion objective using glycerol immersion media. Overlapping regions (10%) were collected as Z-stacks at a resolution of 541 × 541 × 1 µm. The overlapping Z-stack of a quadrant was stitched using XuvStitch freeware^83^. The confocal Z-stack of the stitched quadrants of the limbus was rendered in three dimensions using the Surpass mode of Imaris 9.2 (Bitplane). Imaris Surface tool was used to render a surface onto the endomucin labeled SC. The volume was obtained from the “Statistic” tab under the surface algorithms in the software. The data was downloaded as a.csv file. The volumes of SC in quadrats were graphed using PlotsOfData-A web app for visualizing data together with their summaries^84^.

### AqH dynamics by Gadolinium magnetic resonance imaging (Gd MRI)

AqH dynamics were analyzed by 3D Gd MRI as described^34,35^. The MR imaging data are accessible via the following url: https://civmvoxport.vm.duke.edu/voxbase/studyhome.php?studyid=733. All MRI measurements were performed utilizing a 7.1-Tesla/22-cm horizontal bore Magnex magnet with an Agilent Direct Drive Console (Santa Clara, CA, USA), providing up to 770 mT/m gradient strength, and a 35 mm transmit–receive birdcage coil. Mice (*n* = 5) were anesthetized with isoflurane (2% for induction and 1.5% for maintenance) and kept warm with warm circulating air during the MRI experiment. Respiration rate was monitored using a small pneumatic pillow (SA Instruments, Inc., Stony Brook, NY, USA). Gadolinium-DTPA (Magnevist, Schering, Germany) was intraperitoneally (i.p.) injected at a dose of 0.3 mmol/kg after one T1-weighted MR image was acquired at baseline. The MR contrast was thus administered at a dose calculated to normalize the body mass for each animal.

Some mice were treated with the IOP lowering compound Ripasudil (0.4% normal isotonic saline; Sigma), which was administered in 5 μl drops to the right eye, while normal isotonic saline was added to the left eye. T1-weighted MR images were acquired using a gradient echo sequence repeated over 2 h, sampling at 10 m intervals, for a total of 12 scans. The imaging parameters were repetition time/echo time = 200/1.92 ms, the field of view = 14.4 × 14.4 mm^2^, matrix 192x192, flip angle 20 degrees, BW 62.5 kHz, and the in-plane resolution was 75 × 75 μm^2^. 11 coronal slices, 0.38 mm thick were acquired. 16 averages were used for each scan, resulting in a total scan time for each temporal sampling interval of 10 min 12 s. An additional scan was acquired at the end of the dynamic contrast-enhanced study, using identical parameters but increasing the number of averages to 64, to help delineate the anatomy. Select specimens were imaged ex vivo using a multi gradient-echo sequence. Eye specimens were prepared after trans cardiac perfusion fixed with a mixture of saline and ProHance (10%), followed by a mixture of formalin and ProHance (10%). The imaging parameters were repetition time/echo time = 50/3 ms, the field of view = 25.6 × 12.8 × 12.8 mm^2^, matrix 512 × 256 × 256, flip angle 60°, BW 62.5 kHz, and the 3D isotropic resolution was 50 μm × 50 μm × 50 μm.

Regions of interest (ROIs) were manually drawn in the T1-weighted imaging slice bisecting the center of the globes, using ImageJ v1.47 (Wayne Rasband, National Institutes of Health, Bethesda, MD, USA). We measured the enhancement of the signal intensity (brightness of voxels) due to Gd accumulation in the AC, and our measurements were averaged over the entire ROIs. To compensate for inter animal variability and to reflect the relative enhancement in each animal, these measurements were normalized to the 10 min baseline. Several studies have demonstrated a linear relationship between the concentration of Gd and the spin-lattice relaxation rate (R1 = 1/T1) over limited ranges of concentration^85–87^. MØrkenborg et al.^88^ have performed experiments at 7 T, the field used in these studies, and demonstrated a linear correlation (*r*^2^ > 0.92) between signal intensity in a gradient echo for concentrations ranging between 0 and 3.0 mmol/lGd-DTPA. Thus, while concentration was not measured directly by the measurement of R1, it can be inferred from the signal intensity. In addition, we have a curve normalization to the same time (10 min) acquired in each animal to adjust for different gains. The averaged time course from each ROI measurement before and after Gd injection was fitted into a sixth-degree polynomial using MatLab2016a (The MathWorks, Inc., Natick, MA, USA). The peak percentage (%) Gd signal enhancement, time to peak, the initial rate of Gd signal increase within the first 10 m after Gd injection, and the area under the curve was extracted from the time courses and compared between both eyes of the same groups using two-tailed paired *t* tests, and across groups using one-way ANOVA and post hoc Tukey’s tests. Data are presented as mean ± standard deviation unless otherwise specified. Results were considered significant when *p* < 0.05.

### Cell lines

Human kidney HEK-293T cells were obtained from ATCC and grown in DMEM plus 10% fetal bovine serum (FBS). Primary HTM cells were provided by Dr. T. Borras. Cells were grown in Modified IMEM (Cat. No. A1048901) supplemented with 10% FBS (Gibco, Grand Island, NY) and 50 μg/ml gentamycin (ThermoFisher) and used at <3 passages. Immortalized HTM-like HTM5 cells (TM5)^90^ were cultured in DMEM/F12 supplemented with 10% FBS, l-glutamine, penicillin (100 units/ml), streptomycin (0.1 mg/ml), and amphotericin B (4 mg/ml). Cells were tested negative for mycoplasma at NIEHS or UCSF.

### Reporter assay

HEK-293T cells were transfected in Opti-MEM with pTAL-Luc-(GLISBS)6 reporter, in which the luciferase reporter is under the control of six copies of GLISBS, pCMV-β-Gal, and a pCMV10 expression plasmid containing wild type Flag-GLIS1 or a Flag-GLIS1 mutant using Lipofectamine 2000. Twenty-four-hour later cells were harvested into 125 μl reporter lysis buffer and luciferase activity and β-galactosidase levels were measured using a luciferase assay kit (Promega, Madison, WI) and a luminometric β-galactosidase detection kit (Takara Bio, Palo Alto, CA) following the manufacturer’s protocol. Experiments were carried out in independent triplicates^89^.

### GLIS1 shRNA knockdown

GLIS1 knockdown in HTM cells was performed by infecting cells with GLIS1 shRNA lentivirus (Dharmacon; GLIS1#1-TRCN0000107705 and GLIS1#5-TRCN0000107709 or scrambled shRNA (control) (MOI 1:10). These cells are referred to as HTM(shGLIS1) and HTM(Scr). Forty-eight hours later cells were collected and RNA isolated with a Purelink RNA mini kit (ThermoFisher Sci., Rockford, IL) for RNA-Seq analysis as described^32,46^.

### Quantitative-PCR

Kidney, ciliary body, TM, cornea, and retina were dissected from eyes of 3-month-old WT mice (*n* = 3), RNA was isolated using a RNeasy mini kit (Qiagen) and reverse-transcribed using the High-Capacity cDNA Archive Kit (Applied Biosystems, Foster City, CA). Glis1 expression was then measured by digital droplet PCR (ddPCR) using the QX200^™^ Droplet Digital^™^ PCR System (BioRad) and normalized to Hsp90a01 expression. Primers for Glis1 (Mouse; PrimePCR ddPCR Expression Probe Assay (BioRad); FAM; dMmuCPE5121630) and Hsp90ab1 (Mouse; PrimePCR ddPCR Expression Probe Assay (BioRad); HEX; dMmuCPE5097465). Accepted ddPCR reads had a minimum of 12,000 events and cDNA concentrations were adjusted to be within 10–10,000 positive events. To analyze gene expression from cultured cells, QPCR analysis was performed using SYBR Green I (Applied Biosystems, Foster City, CA). RNA from cultured HTM and TM5 cells was isolated with a Purelink RNA mini Kit (ThermoFisher) and QPCR analysis was performed as described previously^32,46^. RNAs from human tissues were from a Clontech Human Total RNA Master Panel II (#636643). Primer sequences are listed in Supplementary Table 1.

### ChIP-Seq analysis with Flag-GLIS1‐HA HTM and TM5 cells

Since no suitable GLIS1 antibody is available for ChIP-Seq analysis, we used primary HTM cells transiently expressing doxycycline (Dox)-inducible Flag‐GLIS1‐HA and a TM-like cell line, TM5, that stably expressed Dox-inducible Flag-GLIS1-HA. First, a pIND20(Flag‐GLIS1‐HA) plasmid was generated by inserting Flag‐GLIS1‐HA into the (Dox)-inducible lentiviral expression vector pIND20^91^ and are referred to as HTM(pIND-GLIS1) and TM5(pIND-GLIS1), respectively. Lentivirus was generated by transient transfection of pIND20(Flag‐GLIS3‐HA) in HEK293T cells together with psPAX2 and pMD2.G plasmids. TM5 cells were infected with the pIND20(Flag‐GLIS1‐HA) lentivirus for 48 h and then selected in a medium supplemented with 750 μg/ml G418 (Invitrogen, Carlsbad, CA). Flag‐GLIS1‐HA expression was induced by the addition of 300 μg/ml Dox (Sigma–Aldrich, St. Louis, MO). The expression of Flag-GLIS1-HA protein was examined by immunofluorescence. The relative fluorescent signal in nuclei was determined using ImageJ software (Fuji) as described^92^. To identify genes directly regulated by GLIS1, ChIP-Seq analysis was performed using TM5 cells stably expressing doxycycline (Dox)-inducible GLIS1-HA. ChIP analysis was performed as described previously^32,46^. Cells were treated with and without Dox for 18 h and crosslinked with 1% formaldehyde in PBS for 10 min at RT and then quenched by glycine (final 125 mM) for 10 min at RT. Cells were washed two times with PBS and then sonicated for 40 min (S220 focused-ultrasonicator, Covaris, Woburn, MA). After removal of cell debris, chromatin was incubated overnight with HA antibody (Cell Signaling, #3724) and subsequently, incubated with Dynabeads Protein G (ThermoFisher Scientific, 10004D) for 3 h at 4^o^C to pulldown GLIS1-HA-chromatin complexes. The chromatin-bound beads were then washed and reverse cross-linked. Libraries were made with the ChIPed-DNA using Nextflex ChIP-Seq Library Prep kit (PerkinElmer). Sequencing reading was performed with a NovaSeq 6000 system (Illumina). TM5(-Dox) cells served as a negative control to determine specificity^93^. UCSC Genome Browser Human Feb. 2009 (GRCh37/hg19) Assembly was used to generate the genome browser tracks.

### ChIP-seq analysis

ChIP-seq data were generated as single-end reads with a NovaSeq 6000 (Illumina). Raw sequence reads were filtered to remove any entries with a mean base quality score < 20. Adapters were removed via Cutadapt v1.12 with parameters “-a AGATCGGAAGAG -O 5 -q 0”, then reads were filtered to exclude those with length <30 bp after trimming. Filtered and trimmed reads were mapped against the hg19 reference assembly (excluding haplotype chromosomes) via Bowtie v1.2, with only uniquely mapped hits accepted. Duplicate mapped reads were removed by Picard tools MarkDuplicates.jar (v1.110). Initial peak calls were made with HOMER (v4.10.3) with parameters “-style factor -fdr 0.00001”, comparing each ChIP sample (Dox+ or Dox−) against its associated input sample. The Dox+ peak set was then filtered to exclude any peak that (a) overlapped a Dox- peak, (b) has fold change over input <8× (as reported by HOMER), or (c) has fold change over local signal <8x (as reported by HOMER). The Dox+ peaks were re-sized to 200 bp centered on the called peak midpoints prior to downstream analysis. Enriched motifs were identified by HOMER ‘findMotifsGenome’ at “-size is given” and all other parameters default. Coverage tracks for genome browser views were generated by extending each uniquely mapped non-duplicate read to the estimated average fragment size of 150 bp, depth normalizing to 25 M reads, then converting to bedGraph format with BEDtools v2.24.0 genomeCoverageBed and subsequently to bigwig format with UCSC utility bedGraphToBigWig v4.

### RNA-seq analysis

RNA-seq data was generated as paired-end reads with a NextSeq 5000 (Illumina). Raw sequence reads were filtered to remove any entries with a mean base quality score < 20 for either end in the pair. Filtered reads were then mapped to the hg19 reference assembly (excluding haplotype chromosomes) via STAR v2.5 with parameters “–outSAMattrIHstart 0–outFilterType BySJout–alignSJoverhangMin 8–limitBAMsortRAM 55000000000–outSAMstrandField intronMotif–outFilterIntronMotifs RemoveNoncanonical”. Counts per gene were determined via featureCounts (Subread v1.5.0-p1) with parameters “-s0 -Sfr” for Gencode V28 gene models. Differential analysis was performed with DESeq2 v1.14.1.

### Pathway analysis

Pathway analysis was performed via DAVID tools (v6.8) for KEGG pathway analysis^94,95^.

### Immunostaining

Expression of GLIS1-βGAL fusion protein was examined by staining tissue sections with chicken anti-βGAL (1:500, ab9361, Abcam) and Alexa Fluor@ 488 donkey anti-chicken IgG (1:2000, A11039, Invitrogen) as described previously^96^. Expression of Anti-HA fusion protein was examined by staining cells with rabbit Anti-HA (1:250; #3724, Cell Signaling Technology) and Alexa Fluor@ 488 donkey anti-rabbit IgG (1:2000, A21208, Invitrogen). Fluorescence was observed in a Zeiss LSM 710 confocal microscope.

### Genetic association analyses

To determine the association of genetic variants in the *GLIS1* region with glaucoma, we utilized the Genetic Epidemiology Research in Adult Health and Aging (GERA) cohort comprising of 4986 POAG cases and 58,426 controls and a multiethnic UK Biobank (UKB; https://www.ukbiobank.ac.uk/) cohort consisting of 7329 glaucomas (subtype unspecified) cases and 169,561 controls from five ethnic groups (European, East Asian, South Asian, African British, and mixed ancestries. The GERA cohort consists of 110,266 adult men and women, 18 years and older, who are of non-Hispanic white, Hispanic/Latino, Asian, or African American ethnicity. Participants from the GERA cohort are members of the Kaiser Permanente Northern California (KPNC) integrated health care delivery system and provided self-reported information via the Research Program on Genes, Environment, and Health (RPGEH) survey. The UKB is a large prospective study following the health of approximately 500,000 participants from 5 ethnic groups (European, East Asian, South Asian, African British, and mixed ancestries) in the UK aged between the ages of 40 and 69. For UKB participants, demographic information and medical history were ascertained through touch-screen questionnaires. UKB participants also underwent a wide range of physical and cognitive assessments, including blood sampling. GERA individuals’ DNA samples were extracted using Oragene kits (DNA Genotek Inc., Ottawa, ON, Canada) at KPNC and genotyped at the Genomics Core Facility of UCSF. DNA samples were genotyped at over 665,000 genetic markers on four race/ethnicity-specific Affymetrix Axiom arrays (Affymetrix, Santa Clara, CA, USA) optimized for European, Latino, East Asian, and African–American individuals. We performed genotype quality control (QC) procedures for the GERA samples on an array-wise basis. Briefly, we included genetic markers with an initial genotyping call rate ≥ 97%, genotype concordance rate > 0.75 across duplicate samples, and allele frequency difference ≤ 0.15 between females and males for autosomal markers. Approximately, 94% of samples and over 98% of genetic markers assayed reached QC procedures. Moreover, genetic markers with genotype call rates < 90% were excluded, as well as genetic markers with a MAF < 1%. We also performed imputation on an array-wise basis. Following the paraphrasing of genotypes with Shape-IT v2.r7271959, we imputed genetic markers from the cosmopolitan 1000 Genomes Project reference panel (phase I integrated release; http://1000genomes.org) using IMPUTE2 v2.3.060. We used the information r2 from IMPUTE2 as a QC parameter, which is an estimate of the correlation of the imputed genotype to the true genotype^9,22,97^. *GLIS1* gene locus was defined as ±500 kb upstream and downstream of the sequence using UCSC Genome Browser Assembly February 2009 (GRCh37/hg19). PLINK v1.9 was used to perform a logistic regression of the outcome and each SNP. Other statistical analyses and data management were performed in the language-and-environment R, version 3.6.0, using functions from the default libraries. All study procedures were approved by the KPNC Institutional Review Board and the protocols followed are compliant with specific Ethical Regulations. Written informed consent was obtained from all participants. The GLIS1 variant-level associations with glaucoma are fully disclosed in the manuscript (Supplementary Data 1 “Association of GLIS1 SNPs with POAG in the multiethnic meta-analysis (GERA+UKB)”). The meta-analysis GWAS summary statistics of glaucoma are available from the NHGRI-EBI GWAS Catalog study https://www.ebi.ac.uk/gwas/search?query=GCST006065.

### Statistical analysis

Data are presented as mean ± standard deviation and were analyzed using a two-tailed Student’s *t* test using Microsoft Excel and/or Prism 8.4 (GraphPad). To identify genetic variants in *GLIS1* associated with glaucoma, we performed logistic regression analysis adjusted for age, sex, and ancestry principal components.

### Reporting summary

Further information on research design is available in the Nature Research Reporting Summary linked to this article.

## Supplementary information

Supplementary information

Description of Additional Supplementary Files

Supplementary Data 1

Reporting Summary

## Data Availability

Source data for Figures [Figs. 1–4 and Supplementary Figs. 1, 2, 5–7, 10–12] are provided with the paper. The ChIP-seq and RNA-seq data described in this manuscript have been deposited in the NCBI Gene Expression Omnibus (GEO) with accession GSE156846. The meta-analysis GWAS summary statistics of glaucoma are available from the NHGRI-EBI GWAS Catalog (https://www.ebi.ac.uk/gwas/downloads/summary-statistics), study accession number GCST006065. The MR imaging data will be accessible after registration at the following URL (a password will be issued the following day that will provide access): https://civmvoxport.vm.duke.edu/voxbase/studyhome.php?studyid=733. Source data are provided with this paper.

## Source data

Source Data
