# Supplementary material for: GLIS1 regulates trabecular meshwork function and intraocular pressure and is associated with glaucoma in humans
Source: Nat Commun. 2021 Aug 12;12:4877. doi: 10.1038/s41467-021-25181-7 (PMC8361148; doi:10.1038/s41467-021-25181-7)
Supplement: Supplementary file 1 — Supplementary information [file 41467_2021_25181_MOESM1_ESM.pdf]

SUPPLEMENTARY FILES

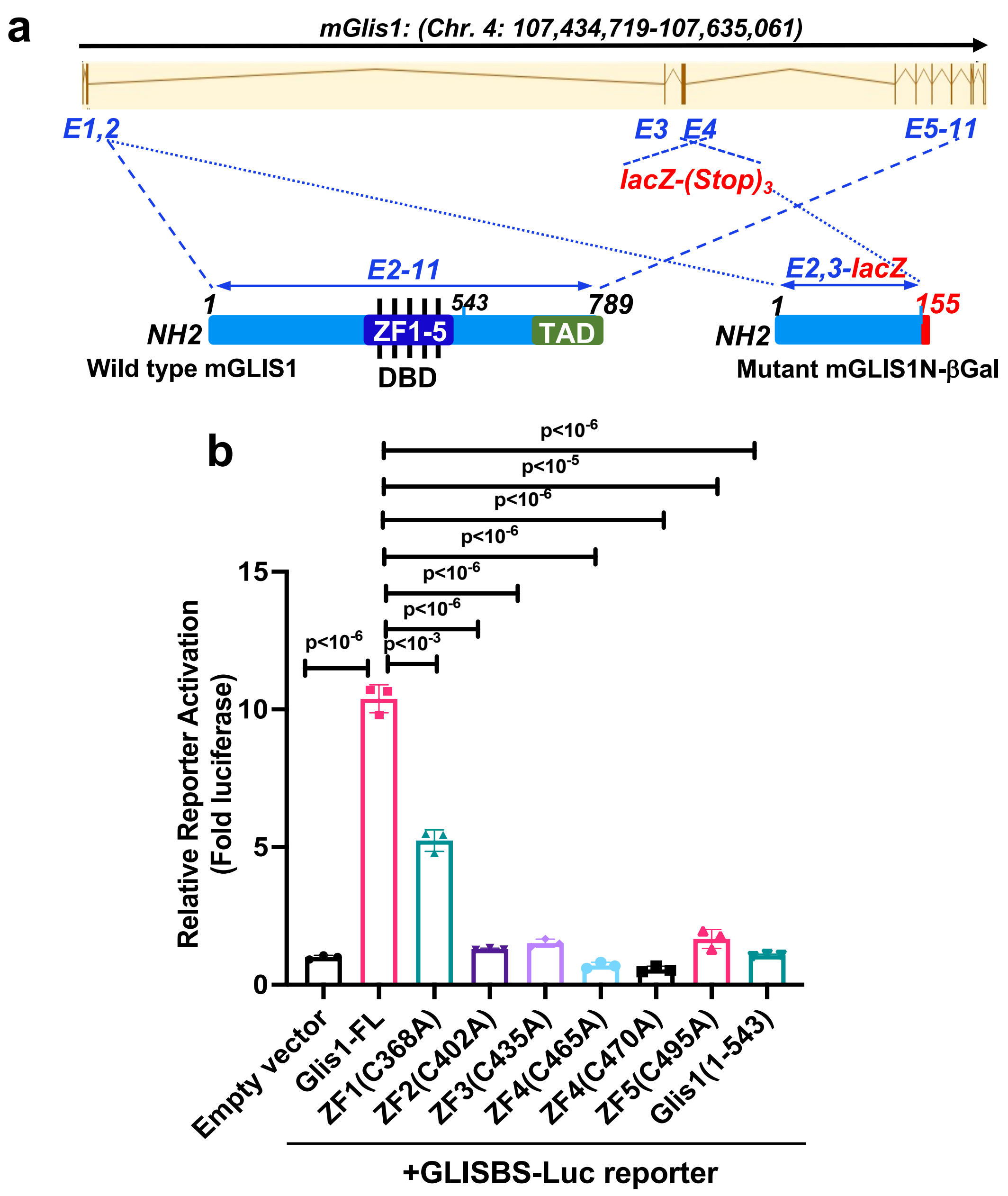

Supplementary Figure 1a, b.

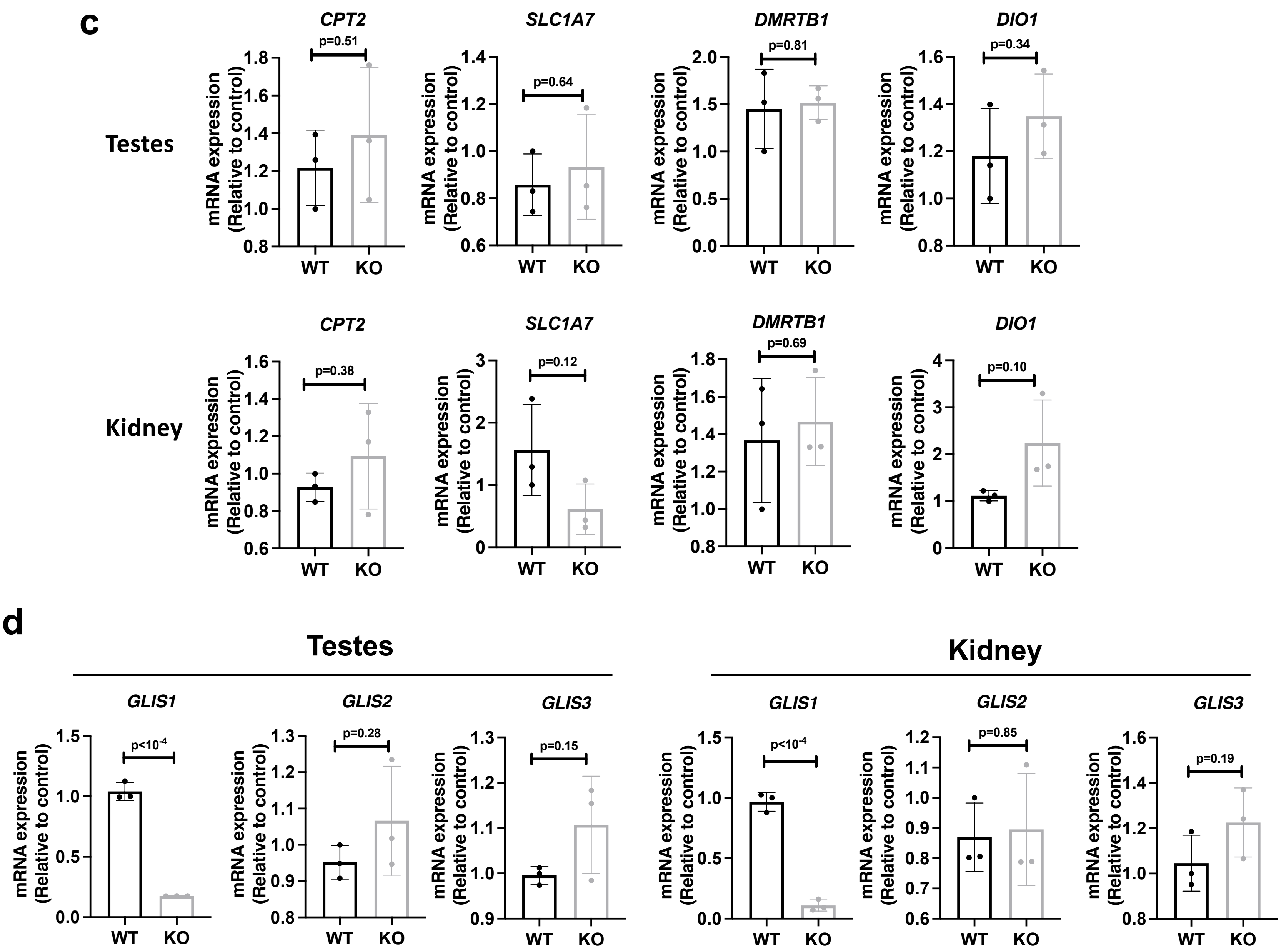

Supplementary Fig. 1c, d

Supplementary Figure 1. a. Genomic map of the mouse *Glis1* gene. In *Glis1*-KO mice 840bp of exon 4 is replaced by lacZ-neo cassette (lacZ-(Stop)3) resulting in the deletion of the DBD and TAD as indicated. This generates a GLIS1ΔC-βGal fusion protein. **b.** The ZF motifs (DBD) and TAD are critical for Glis1 transcriptional activity. The effect of ZF mutations and loss of the TAD on GLIS1 transcriptional activity was examined in HEK293T cells co-transfected with β-Gal and GLISBS-LUC reporters, and the expression plasmid indicated. The relative Luc reporter activation was determined and plotted (n=3, independent replicates). **c, d**. Deletion of exon 4 in *Glis1*-KO mice had no significant effect on the expression of the *Glis1* neighboring genes, *Dmrtb1, Slc1a7, Dio1,* and *Cpt2* (**c**), nor the expression of *Glis2* and *Glis3* (**d**) in *Glis1*-KO kidneys and testes. Data in **b-d** are represented as means ± SD. Statistical analyses were performed with two-tailed Student's t-test. P-values are indicated above bars.

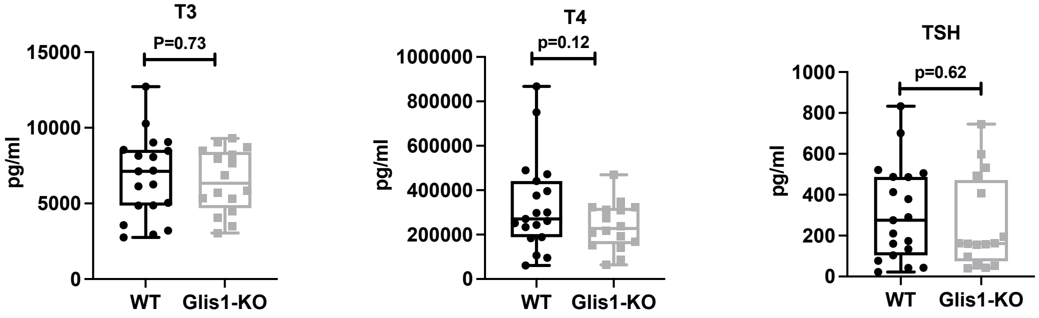

Supplementary Figure 2. *Glis1*-KO eye phenotype is not related to Graves' disease. Blood T3 and T4, and TSH levels in 3 months old WT (n=19 mice) and *Glis1-KO* mice (n=16 mice) were assayed. Data are presented as box plots with quartiles and ranges. Statistical analyses were performed with two-tailed Student's t-test. Median, upper, and lower quartile values: for T3 in WT (7125, 8544, 4860); KO (6339, 8405, 4688); T4 in WT (270415, 441439, 188291); KO (227577, 320409, 156793); TSH in WT (275, 487, 104); KO (161.5, 472, 74.25). Confidence intervals (CI) for T3, T4, and TSH were all 95%.

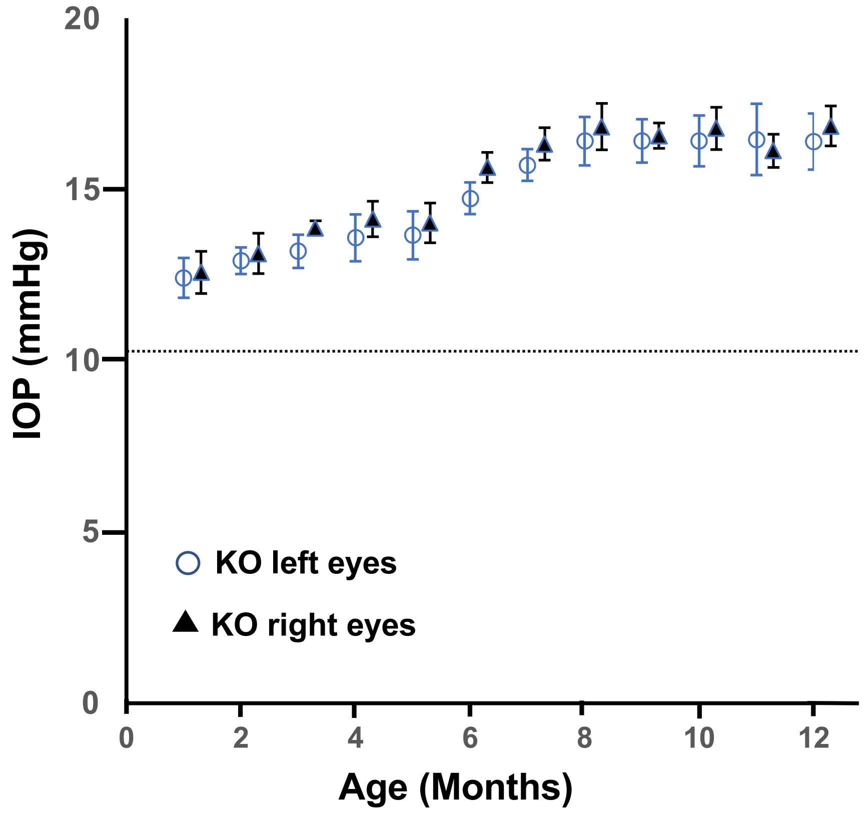

Supplementary Figure 3. Comparison of IOP levels between left and right eyes of male *Glis1*-KO mice as function of age. IOP levels in left and right eyes were measured. Number of mice examined: at 1 and 2 months (n=3); 3 (n=5); 4 and 7 months (n=8); 5 (n=6); 6, 11, and 12 months (n=7)(4 IOP measurements/eye/timepoint). Dotted line indicates basal IOP level in 1-3 months old male WT mice. Data are represented as means ± SD. Statistical analyses were performed with two-tailed Student's t-test. No significant statistical differences were found between right (triangles) and left (circles) eyes. p values at 1 to 12 months are 1.0, 0.91, 0.12, 0.44, 0.76, 0.044, 0.29, 0.75, 0.36, 0.94, 0.13, 0.87, respectively.

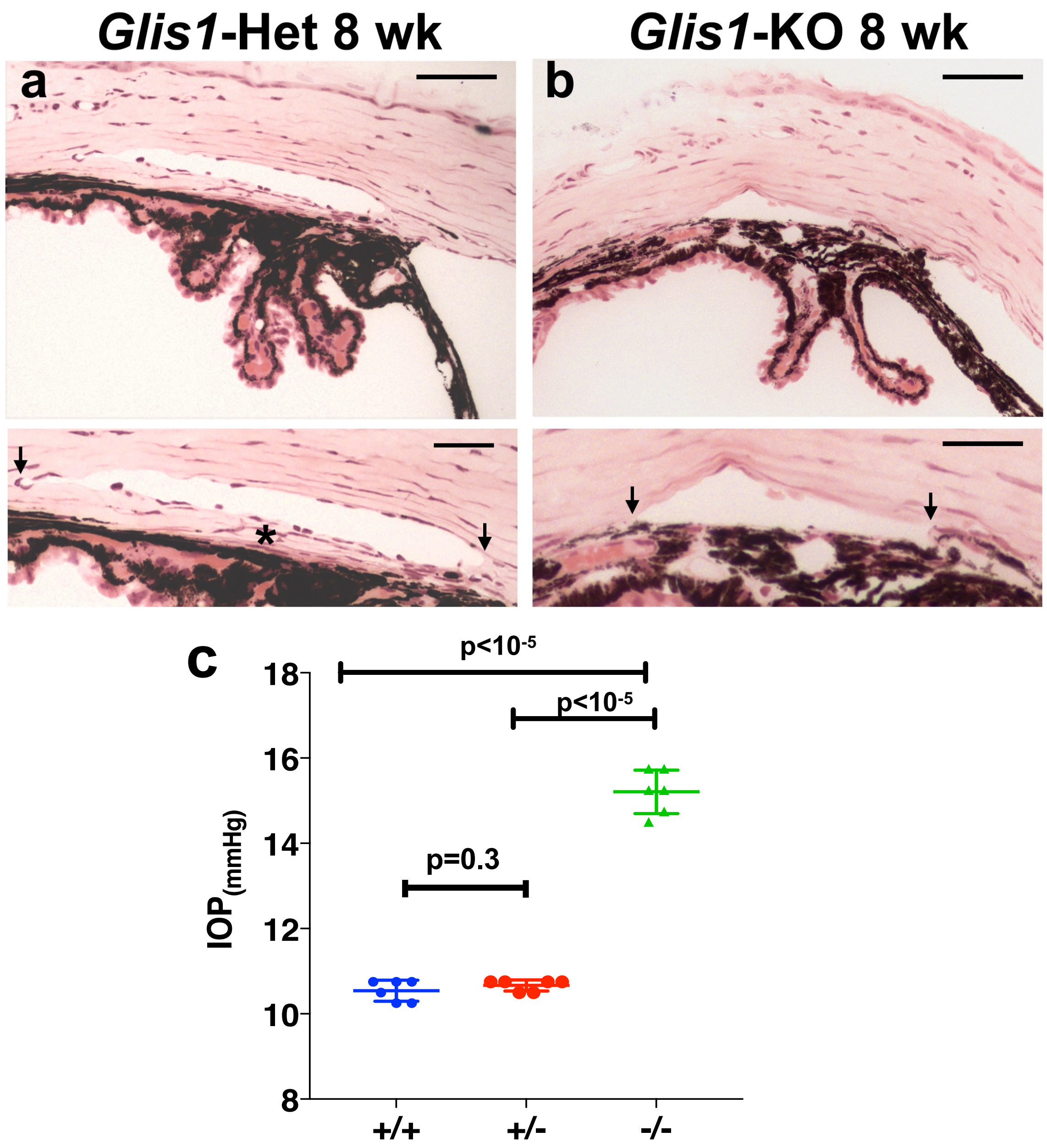

Supplementary Figure 4. ***Glis1*-heterozygous mice exhibit a well-developed ocular drainage structure.**

a, b. Representative histological images of the ocular angle structure from 8-weeks-old *Glis1*-heterozygous **(a)**

and *Glis1*-KO mice **(b)** maintained in C57BL/6NCrl background. *Glis1*-heterozygous eyes showed a well-developed SC and TM (*). In contrast, age-matched *Glis1*-KO eyes exhibited substantial thinning of the TM. Arrows show edges of the SC. The histological assessment was performed on 6 eyes for each genotype with similar results. Scale bar = 50 μm in upper panel and 25 μm lower panel of images in **a** and **b**. **c.** IOP comparison. No significant difference in IOP was observed between 6 months-old WT and heterozygous mice, while homozygous *Glis1*-KO mice showed significantly elevated IOP. Data are represented as means ± SD (p=0.3; 6 eyes each, IOP measured four times in each eye). Statistical analyses were performed with two-tailed Student's t-test.

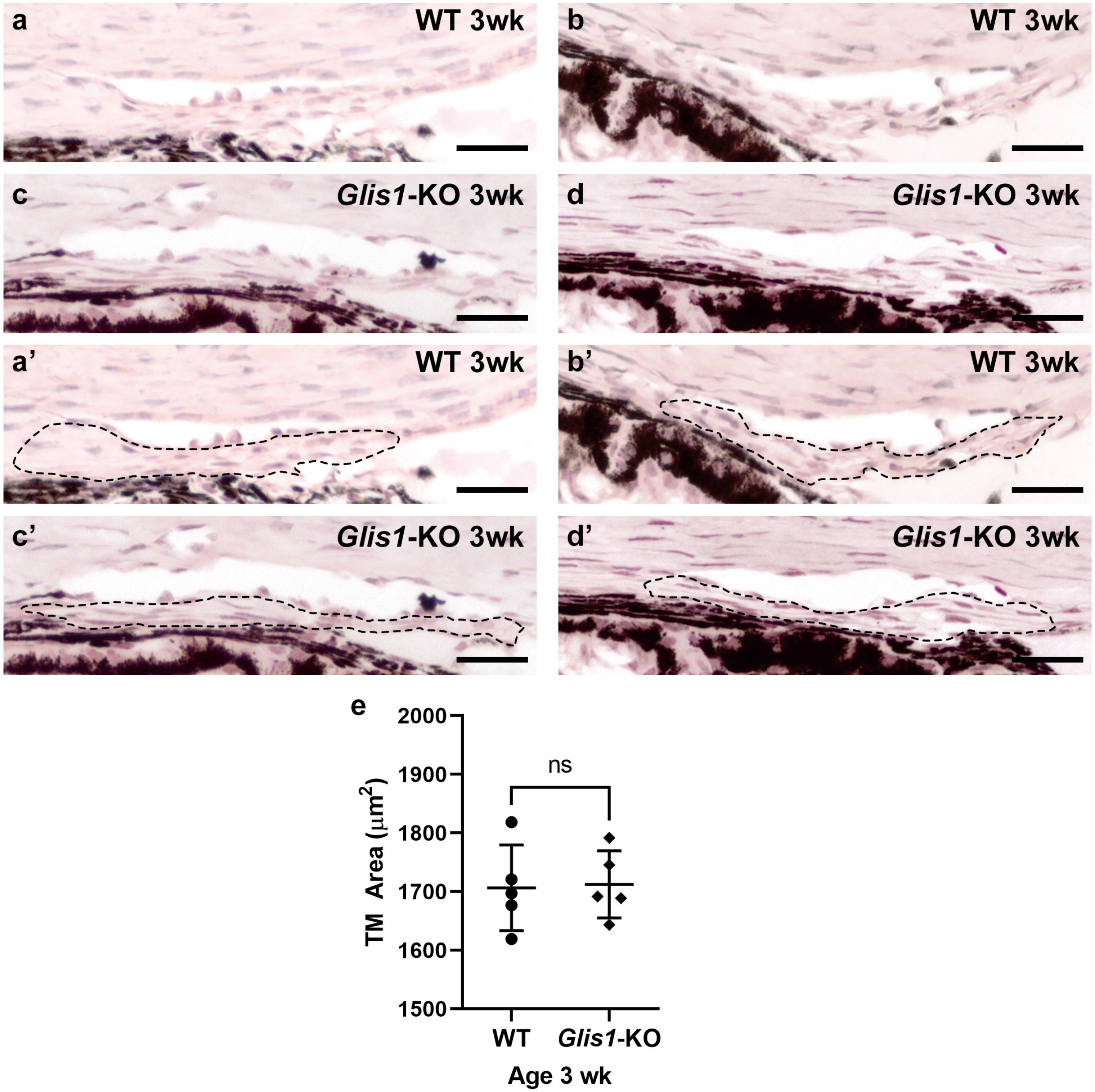

Supplementary Figure 5. Comparison of the TM status of WT and *Glis1*-KO mice. Representative histological images of the ocular angle structure from 3 weeks old WT (**a,b**) and *Glis1*-KO (**c,d**) mice. Lower panel shows the region selected for measuring the TM area in WT (**a',b'**) and *Glis1*-KO (**c',d'**). Images from three

different mice are shown. **e**. Scatter plot showing TM area of 3 weeks-old WT and *Glis1-KO* mice (n = 5 eyes per group). Scale bar = 25 μm. Statistical analyses were performed with two-tailed Student's t-test. At 3 weeks of age, the TM area in *Glis1*-KO is not significantly (ns) different from that in WT mice (p=0.87).

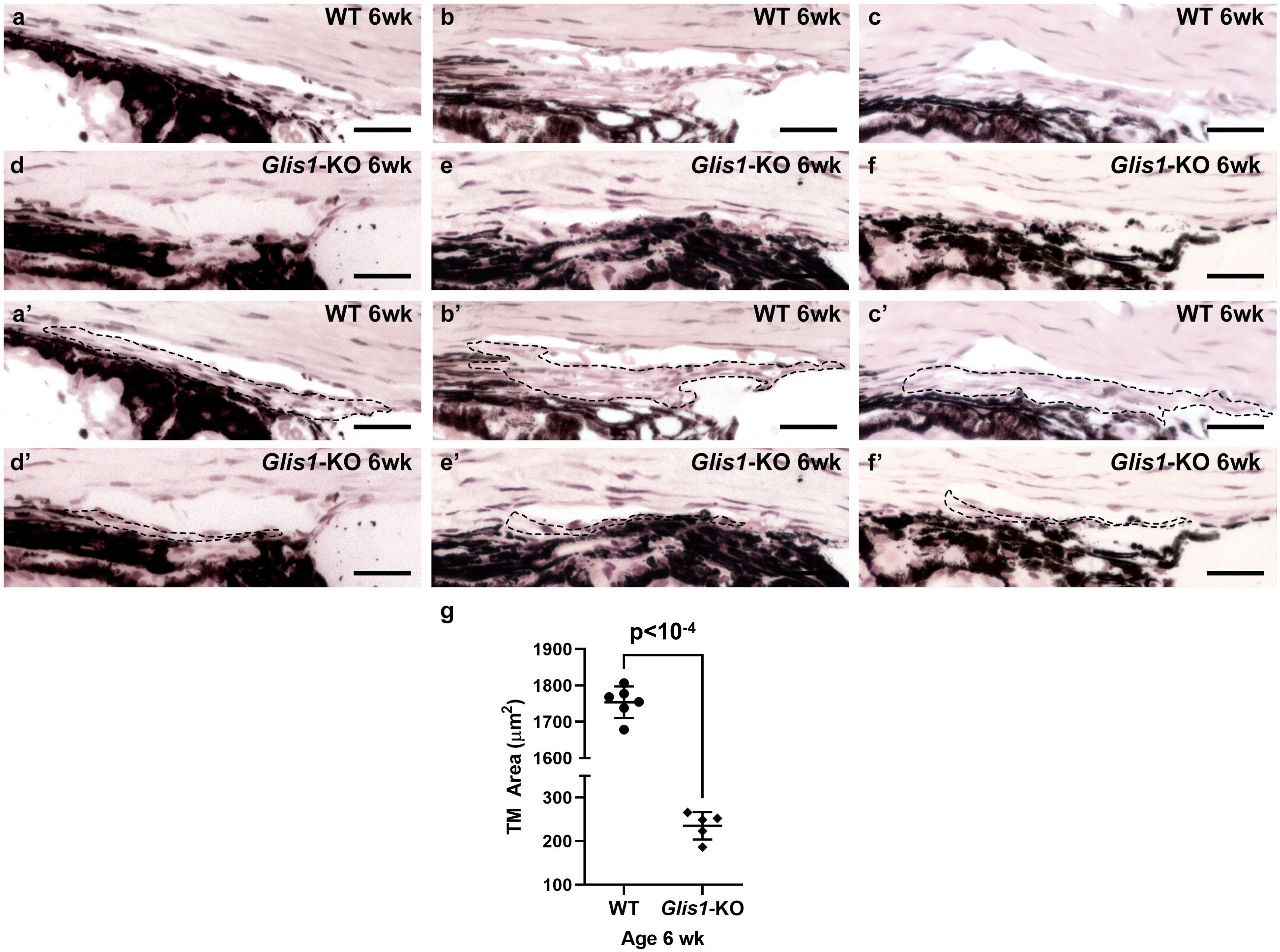

Supplementary Figure 6. TM is substantially thinner in *Glis1*-KO mice at 6 weeks of age. Representative histological images of the ocular angle structure from 6 weeks-old WT (**a,b,c**) and *Glis1*-KO (**d,e,f**) mice. The region selected for measuring the area of the TM is outlined in WT (**a’,b’,c’**) and *Glis1*-KO (**d’,e’,f’**) mice are

shown in the lower panel. Images from three different mice are shown. **g.** Scatter plot showing TM area of 6 weeks-old WT and *Glis1-KO* mice (n = 5 eyes per group). The TM area in *Glis1*-KO is significantly smaller compared to that in WT mice. Statistical analyses were performed with two-tailed Student's t-test. Data are represented as means ± SD. $p<10^{-4}$. Scale bar = 25 μm.

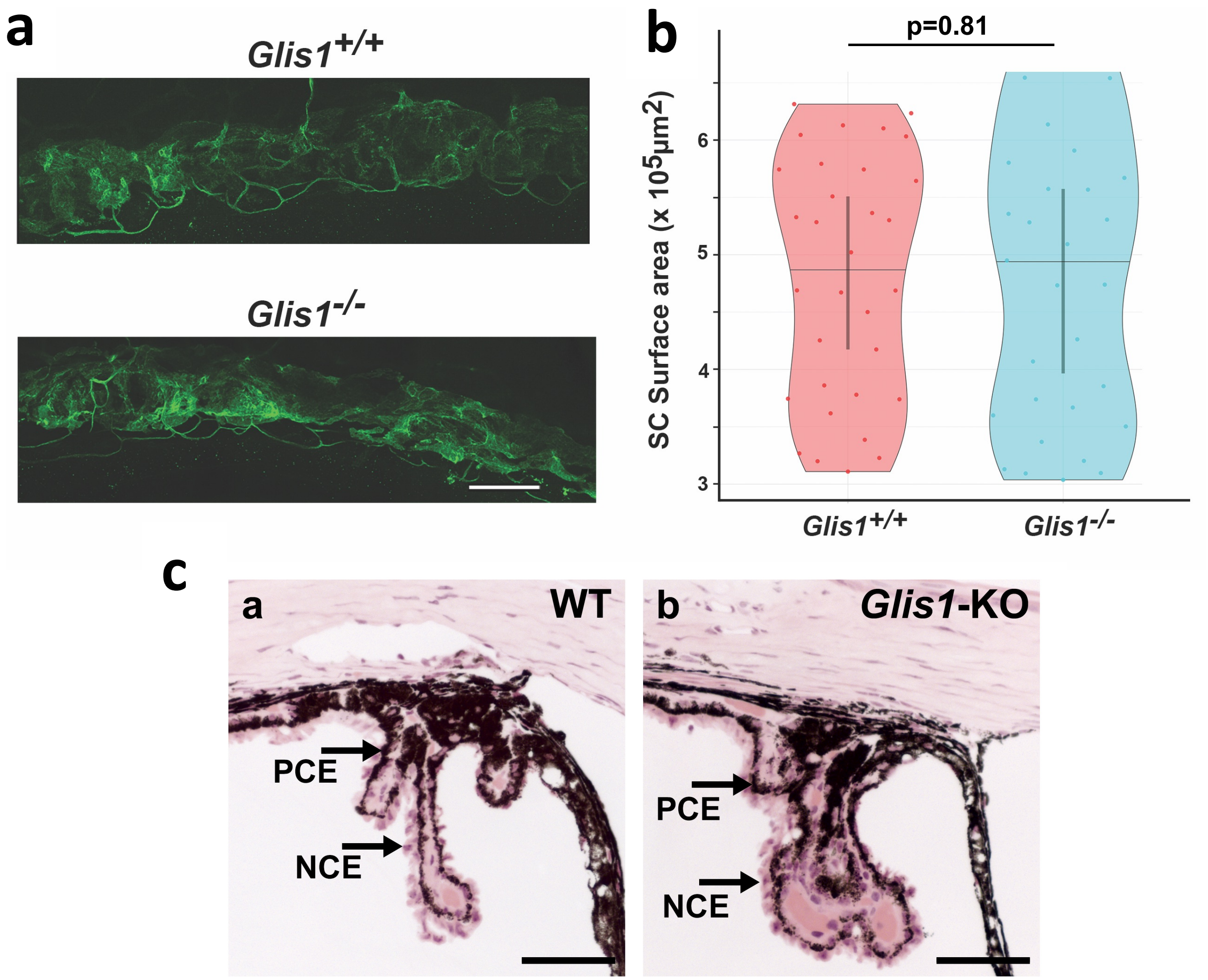

Supplementary Figure 7. The organization of SC and ciliary body is unaffected in GLIS1 null mice. **a**. Representative image of endomucin stained SC from a WT control mouse and *Glis1*-KO mouse at 4 weeks of age. No significant difference was observed. **b**. Quantification of volume of SC in control and *Glis1*-KO mice show no significant difference. Endomucin stained SC volume was computed using Imaris. Graph is mean and 95% confidence interval (CI) of mean. Each dot is the surface of SC in a quadrant of an eye (4 quadrants/eye). In total eight eyes per genotype were measured (a total of 32 measurements per genotype). Statistical analyses were

performed with two-tailed Student's t-test. The volume of SC was not significantly different between *Glis1*-KO and wild type mice (p=0.81). Scale bar = 100μm. **c.** Representative histological image of the ciliary body of 3 months-old wild type and *Glis1*-KO mice showing normal organization of the pigmented ciliary epithelium (PCE) and nonpigmented ciliary epithelium (NCE). Scale bar = 50 μm.

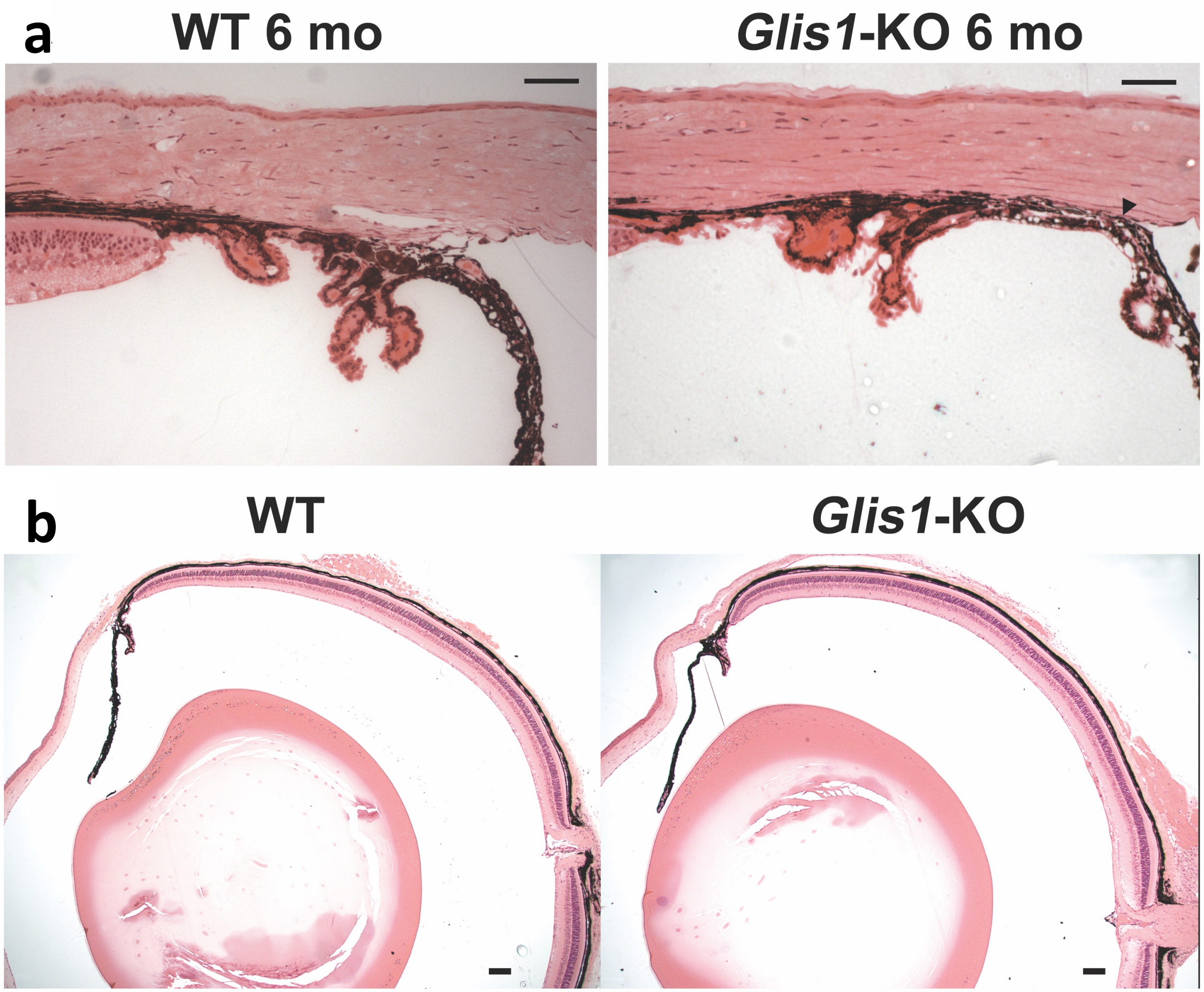

Supplementary Figure 8. a. Ocular angle drainage structures of *Glis1*-KO mice at older ages. The WT control mice exhibit prominent SC and TM. In contrast, *Glis1*-KO mice older than 6 months typically show no visible ocular drainage structures and exhibit synechiae characterized by fusion of the iris and cornea resulting in angle closure. Histological assessment of 6 eyes per group was performed with similar results. Scale bar = 50 µm. **b. Histological analysis of whole WT and *Glis1*-KO eyes.** Representative H&E stained ocular sections from WT and *Glis1*-KO eyes showing that *Glis1*-KO eyes do not exhibit gross morphological abnormalities besides ocular drainage tissue defect compared to the control eyes. Ten eyes per experimental group were assessed with similar results. Scale bar = 100 µm.

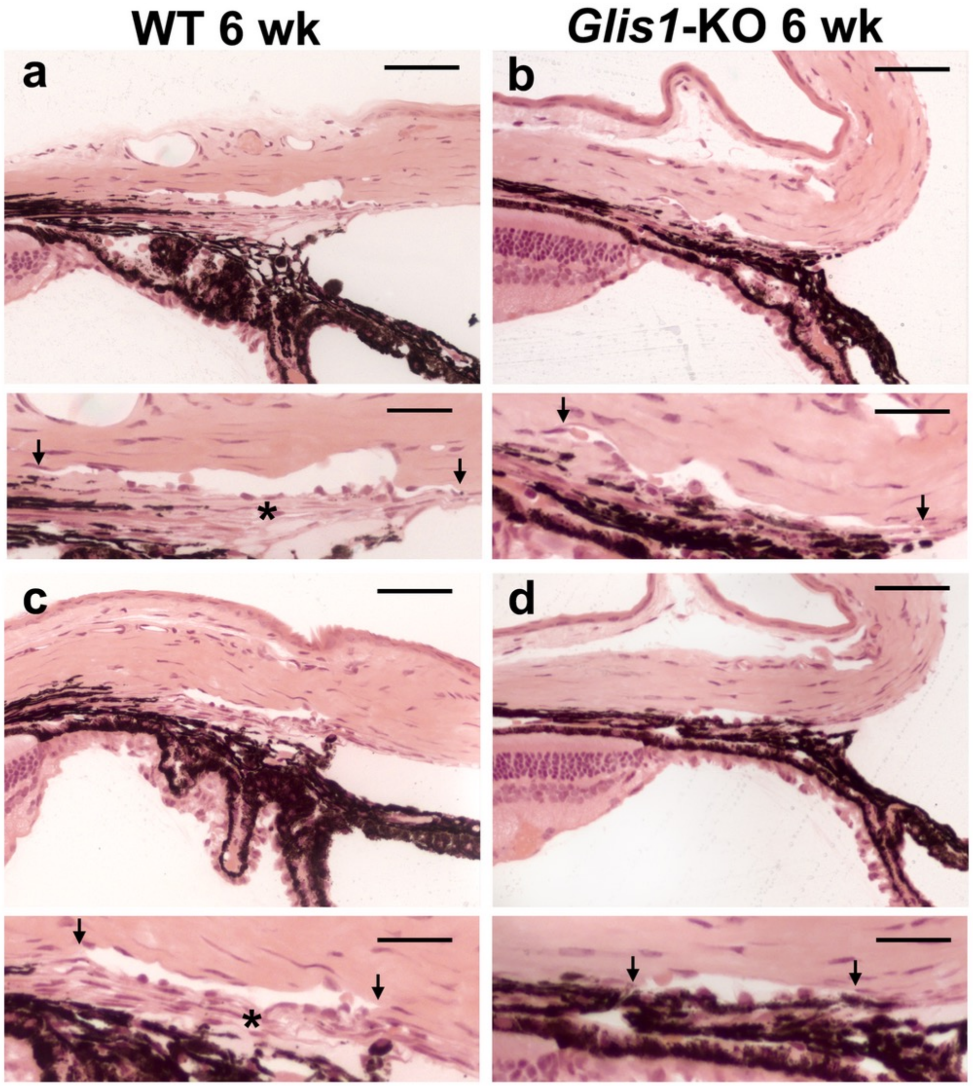

Supplementary Figure 9. Disruption of the ocular angle drainage structures in *Glis1*-KO mice in 129S6/SvEvTac background. a-d. Representative histological images of 2 months old WT mice *Glis1*-KO mice maintained in 129S6/SvEvTac genetic background. **a, c.** WT eyes showed a well-developed SC and TM (*). **b, d.** *Glis1*-KO eyes exhibit hypoplastic TM characterized by substantial thinning of the TM. A magnified version of the image in the upper panel is shown in the lower panel. Arrows show edges of the SC. Eight eyes in each of the experimental groups were histologically assessed with similar results. Scale bar = 50 μm for images in the upper panel and 25 μm for images in the lower panels.

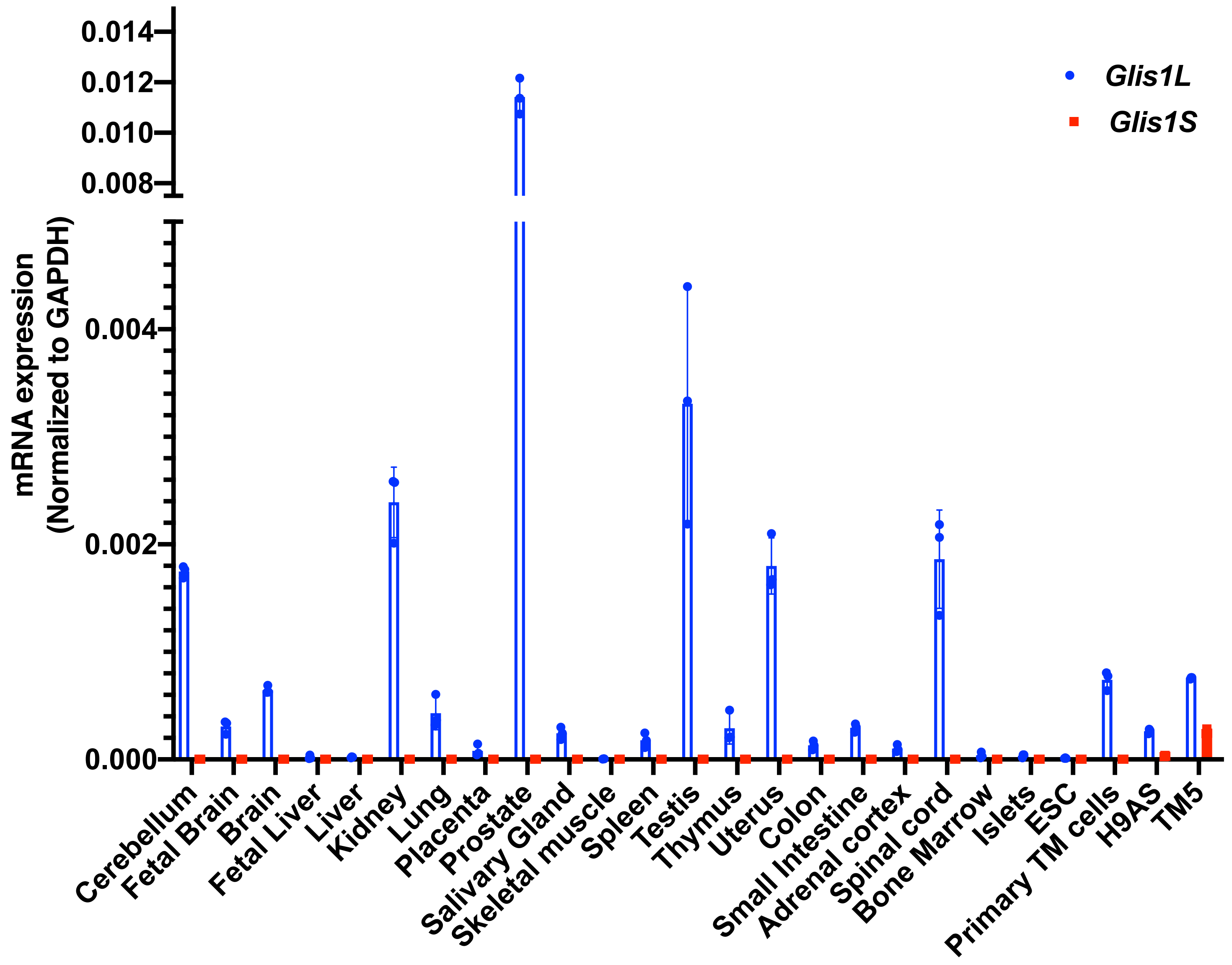

Supplementary Figure 10. Abundancy of the long and short GLIS1 transcripts ($GLIS1_L$ and $GLIS1_S$) in several human tissues and primary human TM cells. mRNA expression (ΔΔCt method) were analyzed by QPCR (n=3, PCR replicates), normalized to housekeeping gene (GAPDH). ESC, human embryonic stem cells. Data are represented as means ± SD.

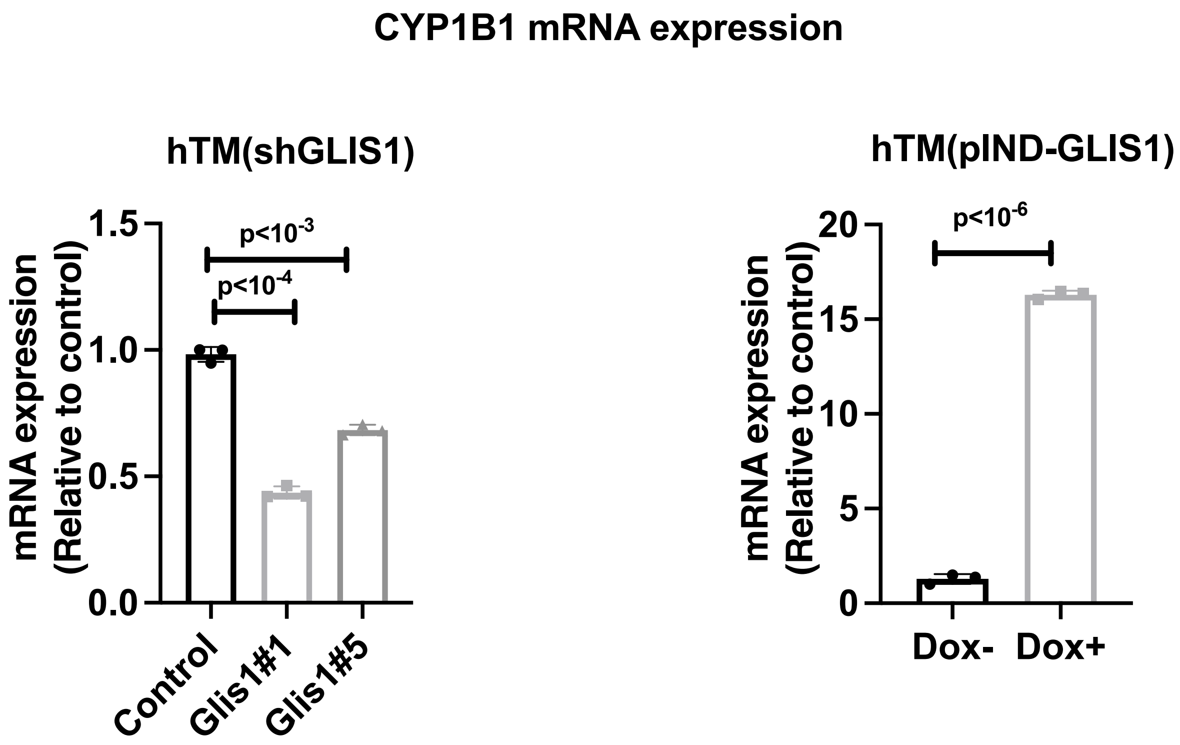

Supplementary Figure 11. GLIS1 regulates *CYP1B1* expression in primary HTM cells. QPCR analysis of CYP1B1 mRNA expression in HTM(shGLIS1) and HTM(Scr) (Control) cells (left panel) and in HTM(pIND-GLIS1) cells (right panel). Statistical analyses were performed with two-tailed Student's t-test. Data are represented as means ± SD (n=3 distinct replicates). P-values are indicated above bars.

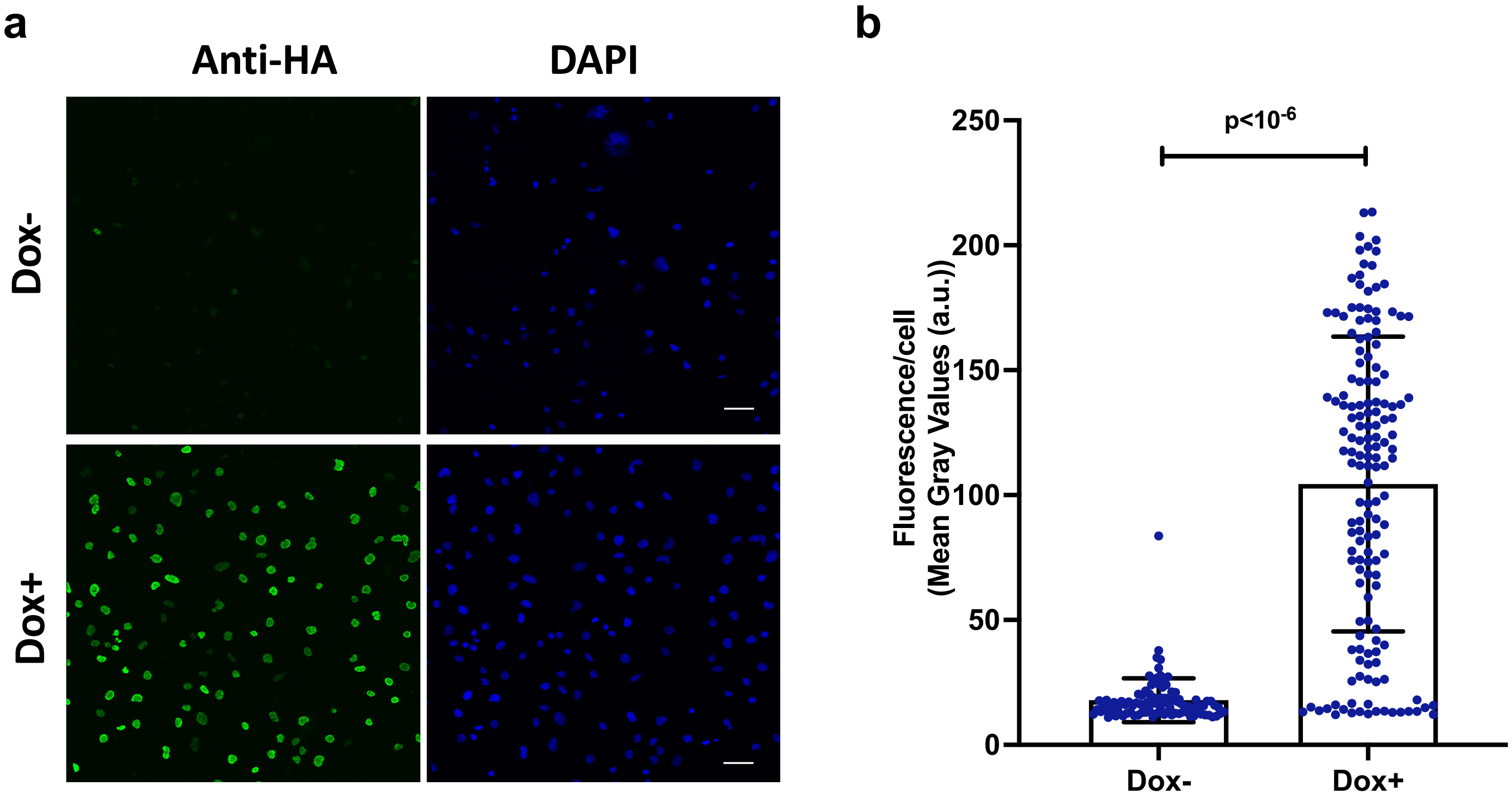

Supplementary Figure 12. Subcellular localization of GLIS1 protein in TM5 expressing Dox-inducible Flag-GLIS1-HA. a, b. Cells were treated for 18 h with and without Dox and then GLIS1-HA protein was visualized by immunofluorescence staining in a confocal microscope (Scale bar = 50 μm). Nuclei were stained with DAPI. The level GLIS1-HA fluorescent signal/cell (-Dox, n=97 cells; +Dox, n=152 cells; each one dish) relative to background between cells treated with or without Dox was determined as described[92]. The significantly higher GLIS1-HA fluorescent signal in Dox-treated cells is consistent with the induction of Flag-GLIS1-HA protein. Statistical analyses were performed two-tailed Student's t-test. Data are represented as means ± SD. P-value is indicated above scatter plots.

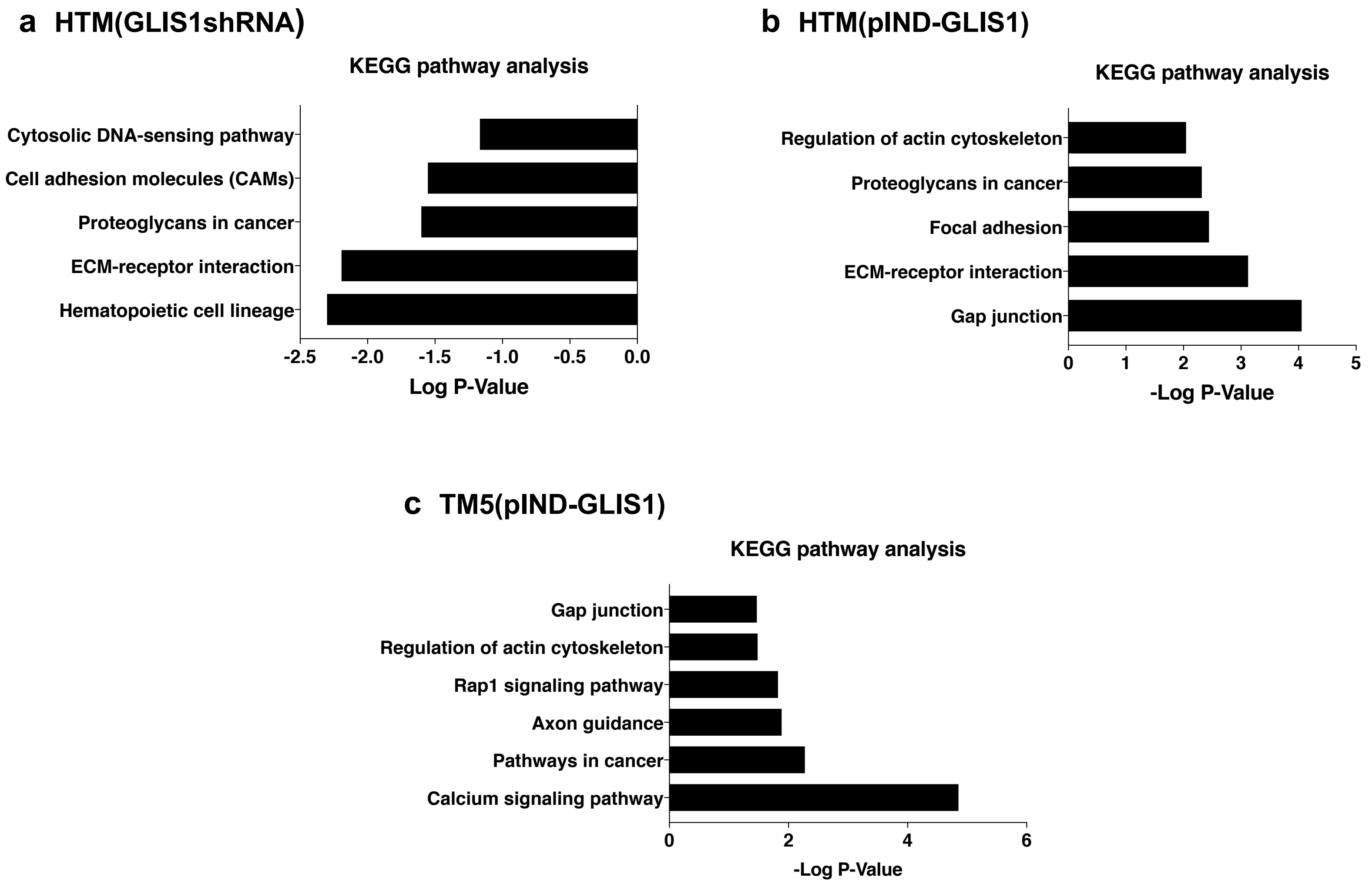

Supplementary Figure 13. KEGG pathway analysis of GLIS1 target genes down-regulated in HTM(GLIS1shRNA) cells (a) and genes up-regulated in HTM(pIND-GLIS1) (b) and TM5(pIND-GLIS1) (c) cells. Top pathways are indicated.

.

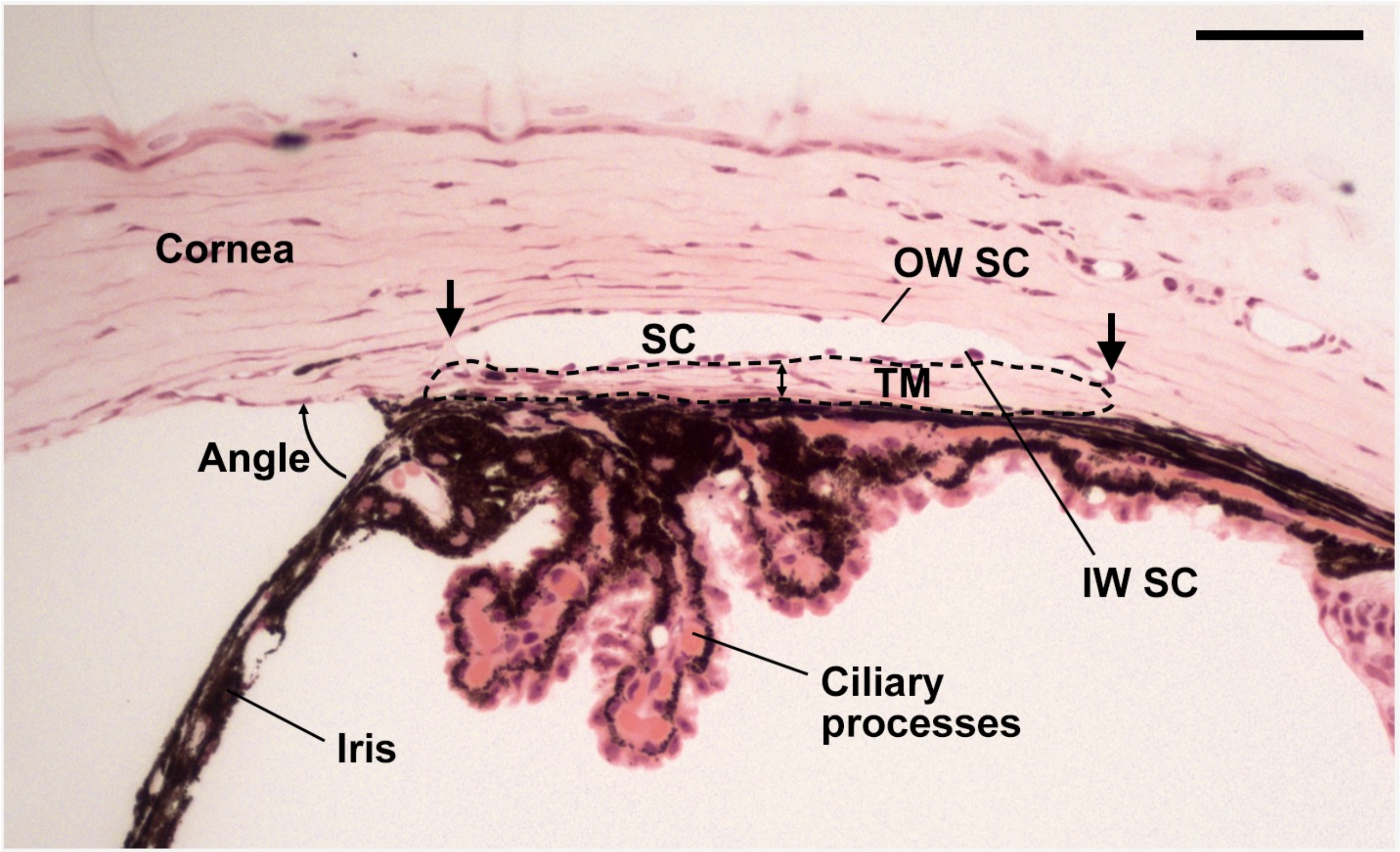

Supplementary Figure 14. Prominent angle relevant tissues are shown in a histological section of the eye. The ocular angle relevant structures, TM, SC, Iris, ciliary processes, inner and outer wall of SC (IW SC, OW SC) are shown to aid the reader. Region selected for measuring the TM area is marked by dotted outline. Scale bar = 50 µm.

Gene	Primer Sequence (5'=>3')
Human	
MYOC	F: CAAGTCAGTTCTGGAGGAAGAG
MYOC	R: CTTCTCAGCCTTGCTACCTC
GLIS1	F: GCCTCTGCCCAGCCCACAAG
GLIS1	R: CCCACCGGCTGCTGGTTCAG
GLIS1 S	F: GTACCACCTGGTTGAATTTCTTCA
GLIS1 S	R: GTGTCCGTAGCTCCCATTCA
GLIS1 L	F: CAGTCCCCGAGACTACGGTC
GLIS1 L	R: GTCCAGCAGGCTCTTCTCTGA
Col8A1	F: AAGTACATCCAGCCCATGC
Col8A1	R: CCATGGAGTCCTGGCTTTC
LOXL4	F: CGTGGCTACCTTTCTGAAACT
LOXL4	R: TATGCTGCTTGGCACTGG
LTBP2	F: GACAACAGCAAACAGCACCAA
LTBP2	R: AGCGGCAGACACAGAGC
APOD	F: AGAGGGACAAGCATTTCATCTT
APOD	R: TCTCAAAGGTTGTTGGGATCTT
BMP2	F: CTGTATCGCAGGCACTCAG
BMP2	R: CCACTCGTTTCTGGTAGTTCTT
CYP1B1	F: GGCCACTATCACTGACATCTT
CYP1B1	R: CCACGACCTGATCCAATTCT
GAPDH	F: CCCATCACCATCTTCCAGGAG
GAPDH	R: CTTCTCCATGGTGGTGAAGACG
Mouse	
Glis1	F: TTCAGCAACTCCAGCGACC
Glis1	R: TTGGAGCAGCCAGGGATCTGA
Gapdh	F: CACATCCCAAAGCCCTCG
Gapdh	R:CTCAGTCCCCTCCTCAGC
Dio1	F: ATCCAGTACTTCTGGTTTGTCC
Dio1	R: CCTGCTGCCTTGAATGAAATC
Dmrtb1	F: GCTTTGCTACCCGGATCA
Dmrtb1	R: TCAGGATTCCCAGCTCCTA
Slc1a7	F: CTGCCTCCTTGGCTTCTTC
Slc1a7	R: CATCTTCAGCATCCGCATCA
Cpt2	F: AGTATCTGCAGCACAGCATC
Cpt2	R: CTGTGCACTGAGGTATCTCTTC
Glis2	F: GCCTGAACAGGATGCTCGAT
Glis2	: CTTCTCACCTGTGTGGGAGC
Glis3	F: AAGTGGAACAGCTGCTGGG
Glis3	R: GAGTGGAGGTAACTGGGAGGA

Supplementary Table 1. List of human and mouse primers used in QPCR analysis.

Gene	GLIS1 down-regulation in HTM shGLIS1#1	GLIS1 down-regulation in HTM shGLIS1#5	GLIS1 Overexpression in HTM	GLIS1 Overexpression in TM5	GLIS1 Binding Peak
ADAMTS10	down	down	up	down	+
ALDH3A1	down	down	up	up	+
APOD	down	down	NC	NC	+
ATXN1L	NC	down	down	up	No
BGN	down	down	up	up	No
BMP2	down	down	NC	up	+
CACNA1A	NC	down	NA	up	+
CARD10	down	down	up	down	+
CDKN1A	down	down	NC	up	+
CHAC1	down	down	NC	up	+
CHI3L1	down	down	down	NC	+
COL16A1	down	down	up	up	+
COL17A1	down	down	NC	up	+
COL1A2	NC	down	up	down	No
COL4A1	NC	down	up	NC	+
COL4A2	down	down	up	NC	+
COL6A2	down	down	down	up	+
COL7A1	down	down	NC	up	No
COL8A2	down	down	up	up	No
CTSH	NC	down	NC	down	+
CYP1B1	down	down	up	down	+
EFEMP1	up	up	up	NC	No
EML2	down	down	NC	up	+
FBLN1	down	down	NC	NC	+
FBLN5	down	down	up	up	+
FBN2	down	down	NC	up	+
FLRT2	down	down	NC	up	+
GLIS1	down	down	up	up	+
HMGA1	down	down	down	up	+
IL20RB	NC	NC	NC	NC	+
ITGA3	NC	down	up	up	+
LOXL1	down	down	up	NC	+
LOXL2	NC	down	up	down	+
LOXL3	down	down	up	up	+
LOXL4	down	down	up	up	+
LTBP2	down	down	up	up	+
MMP2	down	down	up	up	+
MYLK	down	down	up	up	+
MYOC	down	down	NC	up	+
PAPLN	NC	down	up	up	+
PODN	NC	down	NC	up	+
PTGES	down	down	up	NC	+
RTN4	up	up	NC	down	+
SAA2	NA	NA	NC	up	+
SDC1	down	down	up	down	+
SDC2	NC	NC	up	up	+
SEMA4B	NC	down	NC	down	+
SPARC	NC	down	up	down	+
TFAP2B	NC	down	NC	NA	No
TGFBR3	down	NC	up	up	+
TIMP3	NC	down	up	up	+

Supplementary Table 2. Association of GLIS1 peaks (ChIP-Seq) with several GLIS1-regulated genes in GLIS1 down-regulated HTM(shGLIS1) and GLIS1-overexpressing HTM and TM5 cells (HTM(pIND-GLIS1) and TM5(pIND-GLIS1).

NC, no statically significant change.
