# Supplementary material for: GLIS1 regulates trabecular meshwork function and intraocular pressure and is associated with glaucoma in humans
Source: Nat Commun. 2021 Aug 12;12:4877. doi: 10.1038/s41467-021-25181-7 (PMC8361148; doi:10.1038/s41467-021-25181-7)
Supplement: Supplementary file 2 — Description of Additional Supplementary Files [file 41467_2021_25181_MOESM2_ESM.pdf]

**Title:** Supplementary Data 1

**Description: Association of GLIS1 SNPs with POAG in the multiethnic meta-analysis GERA+UKB.** (located ±500 kb upstream and downstream of the GLIS1 gene) with POAG. Abbreviations: CHR, chromosome; BP, base pair position; SNP, single nucleotide polymorphism; A1: effect allele; A2: other allele; N, number of valid studies for this SNP; P, fixed-effects p-value; P.R., random-effects p-value; OR, fixed-effects odds ratio; OR.R., random-effects odds ratio; Q, p-value for Cochrane's Q statistic; I, heterogeneity index (0-100). Most prominent GLIS1 SNP is highlighted.
