# Supplementary material for: GLIS1 regulates trabecular meshwork function and intraocular pressure and is associated with glaucoma in humans
Source: Nat Commun. 2021 Aug 12;12:4877. doi: 10.1038/s41467-021-25181-7 (PMC8361148; doi:10.1038/s41467-021-25181-7)
Supplement: Supplementary file 4 — Reporting Summary [file 41467_2021_25181_MOESM4_ESM.pdf]

nature research

Corresponding author(s): NCOMMS-20-10987A
Anton M. Jetten, ORCID # 0000-0003-0954-4445
Last updated by author(s): Mar 6, 2021

Reporting Summary

Nature Research wishes to improve the reproducibility of the work that we publish. This form provides structure for consistency and transparency in reporting. For further information on Nature Research policies, see our Editorial Policies and the Editorial Policy Checklist.

Statistics

For all statistical analyses, confirm that the following items are present in the figure legend, table legend, main text, or Methods section.

n/a	Confirmed	
☐	☒	The exact sample size (n) for each experimental group/condition, given as a discrete number and unit of measurement
☐	☒	A statement on whether measurements were taken from distinct samples or whether the same sample was measured repeatedly
☐	☒	The statistical test(s) used AND whether they are one- or two-sided<br>*Only common tests should be described solely by name; describe more complex techniques in the Methods section.*
☐	☒	A description of all covariates tested
☐	☒	A description of any assumptions or corrections, such as tests of normality and adjustment for multiple comparisons
☐	☒	A full description of the statistical parameters including central tendency (e.g. means) or other basic estimates (e.g. regression coefficient) AND variation (e.g. standard deviation) or associated estimates of uncertainty (e.g. confidence intervals)
☐	☒	For null hypothesis testing, the test statistic (e.g. F, t, r) with confidence intervals, effect sizes, degrees of freedom and P value noted<br>*Give P values as exact values whenever suitable.*
☒	☐	For Bayesian analysis, information on the choice of priors and Markov chain Monte Carlo settings
☐	☒	For hierarchical and complex designs, identification of the appropriate level for tests and full reporting of outcomes
☒	☐	Estimates of effect sizes (e.g. Cohen's d, Pearson's r), indicating how they were calculated

Our web collection on statistics for biologists contains articles on many of the points above.

Software and code

Policy information about availability of computer code

Data collection	NHGRI-EBI GWAS Catalog; multiethnic meta-analysis (GERA+UKB); GEO #GSE156846; https://civmvoxport.vm.duke.edu/voxbase/ studyhome.php?studyid=733
Data analysis	ImageJ v1.47; MatLab; HOMER (v4.10.3); BEDtools v2.24.0; UCSC utility bedGraphToBigWig; STAR v2.5; Subread v1.5.0-p1; DESeq2 v1.14.1; DAVID tools (v6.8); Gencode V28; Cutadapt v1.12

For manuscripts utilizing custom algorithms or software that are central to the research but not yet described in published literature, software must be made available to editors and reviewers. We strongly encourage code deposition in a community repository (e.g. GitHub). See the Nature Research guidelines for submitting code & software for further information.

Data

Policy information about availability of data

All manuscripts must include a data availability statement. This statement should provide the following information, where applicable:
- Accession codes, unique identifiers, or web links for publicly available datasets
- A list of figures that have associated raw data
- A description of any restrictions on data availability

Molecular ChIP-Seq and RNA-Seq data have been submitted to GEO #GSE156846 and imaging data are available at https://civmvoxport.vm.duke.edu/voxbase/ studyhome.php?studyid=733 (which becomes accessible after registration) and GWAS at https:// www.ebi.ac.uk/gwas/downloads/summary-statistics.
Source files for the following Figs are included: Figs. 1-4 and Suppl. Figs 1, 2, 5-7, 10-12.
No restrictions on data availability: civmvoxport.vm.duke.edu/voxbase/ studyhome.php ?studyid= 733 becomes accessible after registration when password is automatically provided the next day.

Field-specific reporting

Please select the one below that is the best fit for your research. If you are not sure, read the appropriate sections before making your selection.

- [x] Life sciences
- [ ] Behavioural & social sciences
- [ ] Ecological, evolutionary & environmental sciences

For a reference copy of the document with all sections, see nature.com/documents/nr-reporting-summary-flat.pdf

Life sciences study design

All studies must disclose on these points even when the disclosure is negative.

Sample size	Depending on the experiments n using independent samples/mice varied from n=3-10 as indicated in the legends and the Source files provided. QPCR three independent samples determined by our previous experience.
Data exclusions	In the shRNA knockdown of GLIS1, two shRNAs #1 and #5 were used, #5 was done with independent replicates; data from shRNA4 were not included because differential expression showed too many outliers.
Replication	IOP and MRI imaging was carried out with different mice (n=3-10). IOP was measured sequentially 4 times in each eye for each mouse left and right eye as indicated in text and Source files. These data were averaged. QPRCR analysis was carried out with 3 independent samples as stated in text and Source files
Randomization	WT, hets and KO mice were randomly taken at different ages and used for IOP, MRI imaging, and histological analyses (analyses were performed in a blinded manner. Bioinformatics and GWAS studies were carried out in an unbiased manner. ChIP-Seq analysis:Raw sequence reads were filtered to remove any entries with a mean base quality score < 20. Adapters were removed via Cutadapt v1.12 with parameters "-a AGATCGGAAGAG -O 5 -q 0", then reads were filtered to exclude those with length <30bp after trimming as stated in M&M. The meta-analysis was performed using standard procedures, The GWAS summary statistics of glaucoma are available from the NHGRI-EBI GWAS Catalog (https://www.ebi.ac.uk/gwas/downloads/summary-statistics).
Blinding	IOP, imaging, and histological analysis were initially performed in a blinded fashion (not knowing which samples where WT, het or KO). RNASeq and ChIP-analysis were carried out independently by the Bioinformatics Group, which performed this analysis in an unbiased manner. GWAS analysis was also performed in an unbiased manner.

Reporting for specific materials, systems and methods

We require information from authors about some types of materials, experimental systems and methods used in many studies. Here, indicate whether each material, system or method listed is relevant to your study. If you are not sure if a list item applies to your research, read the appropriate section before selecting a response.

Materials & experimental systems

n/a	Involved in the study	
[ ]	[x]	Antibodies
[ ]	[x]	Eukaryotic cell lines
[x]	[ ]	Palaeontology and archaeology
[ ]	[x]	Animals and other organisms
[ ]	[x]	Human research participants
[x]	[ ]	Clinical data
[x]	[ ]	Dual use research of concern

Methods

n/a	Involved in the study	
[ ]	[x]	ChIP-seq
[x]	[ ]	Flow cytometry
[x]	[ ]	MRI-based neuroimaging

Antibodies

Antibodies used	HA antibody (Cell Signaling, #3724); Info on beta-Gal, HA, and Alexa Fluor@ 488 donkey antibodies is provided in M&M.
Validation	In Supplemtary Fig. 11: GLIS1-HA was induced in TM5 cells by doxycycline. GLIS1-HA expression (with HA-antibody) was only detectable in cells treated with Dox and not in untreated cells not expressing GLIS1-HA. Also, motif analysis of ChIP-Seq data (carried out with HA antibody) identified GLIS1 binding sites as the top binding site consistent with the conclusion that HA antibody acts specifically on GLIS1-HA

Eukaryotic cell lines

Policy information about cell lines

Cell line source(s)	Primary human trabecular meshwork (hTM) cells and immortalized TM5 cells provided by Terete Borras, Gulab Zode, and Saidas Nair; HEK292 cells were directly obtained from ATCC.

Authentication	hTM cells highly express CYP1b1 and MYOC, markers for hTM
Mycoplasma contamination	Cells were routinely tested for mycoplasma contamination
Commonly misidentified lines (See ICLAC register)	No commonly misidentified lines were used.

Animals and other organisms

Policy information about studies involving animals; ARRIVE guidelines recommended for reporting animal research

Laboratory animals	C57BL/6NCrl Charles River, Wilmington, MA) and 129S6/SvEvTac (Taconic, Rensselaer, NY) backgrounds for at least 7 generations. Male and female mice were used. Further information is provided in M&M.
Wild animals	No wild animals were used.
Field-collected samples	NA
Ethics oversight	All animal protocols followed the guidelines outlined by the NIH Guide for the Care and Use of Laboratory Animals and were approved by the Institutional Animal Care and Use Committee at the NIEHS.

Note that full information on the approval of the study protocol must also be provided in the manuscript.

Human research participants

Policy information about studies involving human research participants

Population characteristics	we utilized the Genetic Epidemiology Research in Adult Health and Aging (GERA) cohort comprising of 4,986 POAG cases and 58,426 controls and a multiethnic UK Biobank (UKB) cohort consisting of 7,329 glaucoma (subtype unspecified) cases and 169,561 controls from five ethnic groups (European, East Asian, South Asian, African British, and mixed ancestries). Males and females.
Recruitment	Genetic Epidemiology Research in Adult Health and Aging (GERA) cohort and multiethnic UK Biobank (UKB) cohort as described in refs 9, 22, and 92
Ethics oversight	The Institutional Review Board of the NIEHS and Kaiser Foundation Research Institute has approved, respectively, the mouse and human study procedures.

Note that full information on the approval of the study protocol must also be provided in the manuscript.

ChIP-seq

Data deposition

- [x] Confirm that both raw and final processed data have been deposited in a public database such as GEO.
- [x] Confirm that you have deposited or provided access to graph files (e.g. BED files) for the called peaks.

Data access links *May remain private before publication.*	The ChIP-seq and RNA-seq data described in this manuscript have been deposited in the NCBI Gene Expression Omnibus (GEO) with accession GSE156846.
Files in database submission	RESULTS FILES (PEAKS FOR CHIPSEQ, COUNTS-PER-GENE FOR RNASEQ):<br>ChIPseq-TM5-Glis1.peaks.bed.gz<br>RNAseq_counts-per-gene.hTM_overexpr.txt.gz<br>RNAseq_counts-per-gene.hTM_shRNA.txt.gz<br>RNAseq_counts-per-gene.TM5_overexpr.txt.gz<br><br>DEPTH TRACKS:<br>ChIPseq-TM5-DoxNeg-Glis1.normDepth.bigWig<br>ChIPseq-TM5-DoxNeg-input.normDepth.bigWig<br>ChIPseq-TM5-DoxPos-Glis1.normDepth.bigWig<br>ChIPseq-TM5-DoxPos-input.normDepth.bigWig<br>RNAseq-hTM_Control_shRNA-rep1.depthNorm.bigWig<br>RNAseq-hTM_Control_shRNA-rep2.depthNorm.bigWig<br>RNAseq-hTM_Control_shRNA-rep3.depthNorm.bigWig<br>RNAseq-hTM_Glis1_Control-rep1.depthNorm.bigWig<br>RNAseq-hTM_Glis1_Control-rep2.depthNorm.bigWig<br>RNAseq-hTM_Glis1_DOXneg-rep1.depthNorm.bigWig<br>RNAseq-hTM_Glis1_DOXneg-rep2.depthNorm.bigWig<br>RNAseq-hTM_Glis1_DOXpos-rep1.depthNorm.bigWig<br>RNAseq-hTM_Glis1_DOXpos-rep2.depthNorm.bigWig<br>RNAseq-hTM_Glis1_shRNA_1-rep1.depthNorm.bigWig

RNAseq-hTM_Glis1_shRNA_4-rep1.depthNorm.bigWig
RNAseq-hTM_Glis1_shRNA_5-rep1.depthNorm.bigWig
RNAseq-hTM_Glis1_shRNA_5-rep2.depthNorm.bigWig
RNAseq-TM5_Glis1_DOXneg-rep1.depthNorm.bigWig
RNAseq-TM5_Glis1_DOXneg-rep2.depthNorm.bigWig
RNAseq-TM5_Glis1_DOXneg-rep3.depthNorm.bigWig
RNAseq-TM5_Glis1_DOXpos-rep1.depthNorm.bigWig
RNAseq-TM5_Glis1_DOXpos-rep2.depthNorm.bigWig
RNAseq-TM5_Glis1_DOXpos-rep3.depthNorm.bigWig

RAW SEQUENCING FILES:
ChIPseq-TM5-DoxNeg-Glis1.fastq.gz
ChIPseq-TM5-DoxNeg-input.fastq.gz
ChIPseq-TM5-DoxPos-Glis1.fastq.gz
ChIPseq-TM5-DoxPos-input.fastq.gz
RNAseq-hTM_Control_shRNA-rep1.1.fastq.gz
RNAseq-hTM_Control_shRNA-rep1.2.fastq.gz
RNAseq-hTM_Control_shRNA-rep2.1.fastq.gz
RNAseq-hTM_Control_shRNA-rep2.2.fastq.gz
RNAseq-hTM_Control_shRNA-rep3.1.fastq.gz
RNAseq-hTM_Control_shRNA-rep3.2.fastq.gz
RNAseq-hTM_Glis1_Control-rep1.1.fastq.gz
RNAseq-hTM_Glis1_Control-rep1.2.fastq.gz
RNAseq-hTM_Glis1_Control-rep2.1.fastq.gz
RNAseq-hTM_Glis1_Control-rep2.2.fastq.gz
RNAseq-hTM_Glis1_DOXneg-rep1.1.fastq.gz
RNAseq-hTM_Glis1_DOXneg-rep1.2.fastq.gz
RNAseq-hTM_Glis1_DOXneg-rep2.1.fastq.gz
RNAseq-hTM_Glis1_DOXneg-rep2.2.fastq.gz
RNAseq-hTM_Glis1_DOXpos-rep1.1.fastq.gz
RNAseq-hTM_Glis1_DOXpos-rep1.2.fastq.gz
RNAseq-hTM_Glis1_DOXpos-rep2.1.fastq.gz
RNAseq-hTM_Glis1_DOXpos-rep2.2.fastq.gz
RNAseq-hTM_Glis1_shRNA_1-rep1.1.fastq.gz
RNAseq-hTM_Glis1_shRNA_1-rep1.2.fastq.gz
RNAseq-hTM_Glis1_shRNA_4-rep1.1.fastq.gz
RNAseq-hTM_Glis1_shRNA_4-rep1.2.fastq.gz
RNAseq-hTM_Glis1_shRNA_5-rep1.1.fastq.gz
RNAseq-hTM_Glis1_shRNA_5-rep1.2.fastq.gz
RNAseq-hTM_Glis1_shRNA_5-rep2.1.fastq.gz
RNAseq-hTM_Glis1_shRNA_5-rep2.2.fastq.gz
RNAseq-TM5_Glis1_DOXneg-rep1.1.fastq.gz
RNAseq-TM5_Glis1_DOXneg-rep1.2.fastq.gz
RNAseq-TM5_Glis1_DOXneg-rep2.1.fastq.gz
RNAseq-TM5_Glis1_DOXneg-rep2.2.fastq.gz
RNAseq-TM5_Glis1_DOXneg-rep3.1.fastq.gz
RNAseq-TM5_Glis1_DOXneg-rep3.2.fastq.gz
RNAseq-TM5_Glis1_DOXpos-rep1.1.fastq.gz
RNAseq-TM5_Glis1_DOXpos-rep1.2.fastq.gz
RNAseq-TM5_Glis1_DOXpos-rep2.1.fastq.gz
RNAseq-TM5_Glis1_DOXpos-rep2.2.fastq.gz
RNAseq-TM5_Glis1_DOXpos-rep3.1.fastq.gz
RNAseq-TM5_Glis1_DOXpos-rep3.2.fastq.gz

Genome browser session
(e.g. UCSC)

UCSC Genome Browser on Human Feb. 2009 (GRCh37/hg19) Assembly

Methodology

Replicates

TM5-DoxNeg-Input
TM5-DoxPlus-IN
TM5-DoxNeg-IP
TM5-DoxPlus-IP

Sequencing depth

ID	raw reads
TM5-DoxNeg-Glis1	87,362,779
TM5-DoxNeg-input	114,275,152
TM5-DoxPlus-Glis1	105,342,499
TM5-DoxPlus-input	86,340,196

passing deduplication

25,758,469	48.7%
75,795,156	85.0%
58,640,525	64.6%
56,722,793	80.7%

Antibodies

HA antibody (Cell Signaling, #3724)

Peak calling parameters

As indicated in M&M: Raw sequence reads were filtered to remove any entries with a mean base quality score < 20. Adapters were removed via Cutadapt v1.12 with parameters "-a AGATCGGAAGAG -O 5 -q 0", then reads were filtered to exclude those with length <30bp after trimming. Filtered and trimmed reads were mapped against the hg19 reference assembly (excluding haplotype chromosomes) via Bowtie v1.2, with only uniquely-mapped hits accepted. Duplicate mapped reads were removed by Picard tools MarkDuplicates.jar (v1.110). Initial peak calls were made with HOMER (v4.10.3) with parameters "-style factor -fdr 0.00001", comparing each ChIP sample (Dox+ or Dox-) against its associated input sample. The Dox+ peak set was then filtered to exclude any peak that (a) overlapped a Dox- peak, (b) has fold change over input <8x (as reported by HOMER), or (c) has fold change over local signal <8x (as reported by HOMER). The Dox+ peaks were re-sized to 200bp centered on the called peak midpoints prior to downstream analysis.

Data quality

As stated in M&M: Enriched motifs were identified by HOMER 'findMotifsGenome' at "-size given" and all other parameters default. Coverage tracks for genome browser views were generated by extending each uniquely-mapped non-duplicate read to the estimated average fragment size of 150bp, depth normalizing to 25M reads, then converting to bedGraph format with BEDtools v2.24.0 genomeCoverageBed and subsequently to bigwig format with UCSC utility bedGraphToBigWig.

Software

HOMER (v4.10.3); HOMER 'findMotifsGenome'; BEDtools v2.24.0 genomeCoverageBed; UCSC utility bedGraphToBigWig;
